# Mechanism of electrothermal acupuncture in alleviating postherpetic neuralgia

**DOI:** 10.3389/fneur.2026.1750292

**Published:** 2026-06-08

**Authors:** Jianjun Chen, Quan Wang, Shiqiao Li, Xi Cheng, Qingxu Yang, Li Zhan

**Affiliations:** Department of Pain Medicine, Yunnan Provincial Hospital of Traditional Chinese Medicine, Kunming, Yunnan, China

**Keywords:** allodynia, capsaicin, electrothermal acupuncture, neuropathic pain, postherpetic neuralgia

## Abstract

**Purpose:**

To investigate the mechanism of electrothermal acupuncture in alleviating postherpetic neuralgia (PHN).

**Patients and methods:**

A rat model of PHN was created with RTX injection, and electrothermal acupuncture (ETA) was applied at trigger points. Pain sensitivity was assessed using the von Frey test for PWT. ATP levels were measured colorimetrically, ROS via DCFH-DA fluorescence, and pro-inflammatory cytokines (TNF-*α*, IL-6, IL-1β) in tissues using ELISA. DEGs were identified through RNA sequencing, followed by GO and KEGG analyses, and PPI networks were constructed to identify core genes. Real-time quantitative PCR (RT-qPCR) for detecting gene expression levels.

**Results:**

ETA significantly increased PWT values, confirming its analgesic effect. Moreover, compared to the PHN group, ETA treatment significantly attenuated RXT-induced increases in the levels of IL-1β, IL-6, and TNF-*α* (*p* < 0.05). It also reversed the RXT-induced excessive ATP elevation and abnormal ROS accumulation in both tissues (*p* < 0.01), with ETA intervention showing particularly marked suppression of ROS in spinal cord tissue. Differential expression analysis identified 822 DEGs in muscle tissue and 333 in spinal cord tissue. The intersection of these datasets yielded 48 candidate genes that were significantly enriched in interferon-mediated innate immune-related pathways. Using the cytoHubba plugin, five core genes—Mx1, Irf7, Rtp4, Oasl2, and Ifit1bl—were identified through multiple algorithmic screenings. RT-qPCR validation confirmed that ETA intervention significantly upregulated the expression levels of Mx1, Irf7, Rtp4, Oasl2, and Ifit1bl (*p* < 0.05).

**Conclusion:**

ETA alleviates tactile allodynia induced by RTX in rats, at least in part by upregulating the expression of type I interferon-related genes (Mx1, Irf7, Rtp4, Oasl2, and Ifit1bl). This upregulation suppresses the central and peripheral inflammatory responses associated with PHN, while also modulating energy metabolism and redox homeostasis in pain-related tissues. These findings offer novel mechanistic insights supporting the use of ETA as an adjunctive therapy for neuropathic pain management.

## 1 Introduction

Postherpetic neuralgia (PHN) is a persistent neuropathic pain condition that typically follows an acute episode of herpes zoster, representing one of the most frequent complications of varicella-zoster virus (VZV) reactivation (1). Its incidence rises with age (2), and approximately 30–50% of patients experience pain lasting more than 1 year (3), substantially impairing their quality of life. Current pharmacological treatments for PHN, such as amitriptyline, desvenlafaxine, and pregabalin—often provide inadequate relief (4), motivating the exploration of complementary and alternative therapies.

PHN is frequently characterized by hyperalgesia and heat hypoesthesia (5). Although inoculation with VZV or herpes simplex virus type 1 (HSV-1) can evoke mechanical hyperalgesia in rodents, these viral infection models generally fail to reproduce the clinically observed hypothermia and entail potential complications such as cutaneous injury and viral dissemination to the central nervous system (6). Studies have demonstrated that injection of resiniferatoxin (RTX) in adult mice induces selective damage to C-fibers, thereby recapitulating the distinctive clinical manifestations of mechanical hyperalgesia and heat hypoalgesia observed in patients with PHN (7). Accordingly, this model is applicable for investigating the pain mechanisms associated with non-viral PHN (8).

Neuroinflammation and mitochondrial dysfunction are intimately linked to chronic neuropathic pain (9–11). Electrothermal acupuncture (ETA), an emerging therapeutic modality that integrates acupuncture with electrothermal stimulation, has exhibited notable efficacy in managing chronic neuropathic pain (12, 13). Moreover, prior studies have established that electroacupuncture intervention can attenuate reactive oxygen species (ROS) levels (14), potentiate antioxidant enzyme activity (15), and suppress the production of inflammatory mediators (16), thus mitigating pain and enhancing quality of life in patients with PHN (17, 18). Notwithstanding these advances, the precise molecular mechanisms by which ETA ameliorates PHN remain to be elucidated. Therefore, the present study employed RTX-induced rats to replicate the clinical mechanical hyperalgesia characteristic of PHN, with the objective of exploring the downstream neuroinflammatory and oxidative pathways through which electrothermal acupuncture (ETA) alleviates this chronic pain condition, thereby providing a theoretical foundation for its clinical translation.

## 2 Materials and methods

### 2.1 Ethical approval

This study utilized 40 SPF male Sprague–Dawley rats (200–220 g; Saiye Bio, China), which were housed under standard conditions (12-h light–dark cycle, 22–25 °C, 40–60% humidity) in accordance with the National Institutes of Health Guide for the Care and Use of Laboratory Animals (GB/T 35892–2018, China). All procedures were approved by the Medical Ethics Committee of The First Affiliated Hospital of Yunnan University of Chinese Medicine and followed the Chinese National Standards for laboratory animal welfare. Approval Number: YD2023-073.

### 2.2 PHN animal model establishment and intervention

After 1 week of acclimatization, rats were randomly divided into four groups (*n* = 10 per group): normal control (Control), model (PHN), sham electroacupuncture (Sham + PHN), and intrafascial electrothermal acupuncture (ETA+PHN). RTX (Sangon Biotech, China) was dissolved in a vehicle consisting of 10% ethanol, 10% Tween-80 to prepare a 40 μg/mL solution. Prior to model induction, 32 rats were randomly selected for baseline measurement of the mechanical paw withdrawal threshold (PWT) using von Frey filaments. These animals then received a single intraperitoneal injection of RTX (200 μg/kg) to establish the PHN model. Mechanical PWT assessment is initiated on day three post-injection. Successful model induction was defined as the PWT levels were significantly lower than those in the control group (19). Only rats meeting this criterion were included in the subsequent experimental cohort; those that did not were excluded. An additional 8 rats, serving as the vehicle control group, received an equivalent volume of the solvent (10% Tween-80 and 10% ethanol in saline) via the same route. ETA intervention began on day 22 post-RTX injection. Rats in the ETA+PHN group received daily 15-min sessions of ETA at myofascial trigger points, involving needle insertion to a depth of 6 mm with 42 °C electrothermal stimulation, for 21 consecutive days. The Sham + PHN group underwent an identical procedure, except that the needle was placed superficially on the skin over the trigger point without insertion. PWT was reassessed on days 4, 7, 11, 14, and 21 post-intervention. On day 44 post-RTX injection, all animals were euthanized via CO_2_ overdose. Muscle tissue from the hyperalgesic site and spinal cord tissue were harvested for subsequent analysis.

### 2.3 Paw withdrawal threshold (PWT) testing

PWT was conducted in rats before modeling (on day 0 of RTX injection) and after modeling on days 3, 7, 10, 14, 17, and 21 following RTX injection, as well as on days 4, 7, 11, 14, and 21 after electrothermal acupuncture (ETA) intervention. Prior to testing, rats were acclimated for 30 min in a transparent chamber (20 × 20 cm) until exploratory behavior subsided. The left hind paw’s plantar region was tested with a set of von Frey filaments (Shanghai Wangyan Instruments Co., Ltd.) that exerted bending forces of 0.6 to 26 g (20). The mechanical pain threshold was defined as the lowest force that elicited a brisk paw withdrawal or licking response. Each rat underwent four trials, and the average value was recorded as the final PWT.

### 2.4 Colorimetric ATP assay

Following the manufacturer’s protocol, quantification of ATP in muscle and spinal cord tissue samples was performed using a commercial kit (A095-1-1, Nanjing Jiancheng Bioengineering Institute). Briefly, tissues were minced and homogenized in cold distilled water (1:9, w/v). The homogenate was boiled, centrifuged, and the supernatant was collected. Reaction reagents were added sequentially, and after incubation for 5–10 min, absorbance was measured at 340 nm using a multimode microplate reader (EnVision, PerkinElmer). ATP content was calculated based on a standard curve and normalized to total protein concentration (mg protein) in the tissue homogenate.

### 2.5 ROS detection by chemiluminescence

Reactive oxygen species levels in muscle and spinal cord tissues were measured with a commercial ROS assay kit (E004-1-1, Nanjing Jiancheng Bioengineering Institute). Tissues were digested to obtain a single-cell suspension, which was then centrifuged and washed. The cells were treated with 10 μM DCFH-DA and kept in the dark for 30 min. Following washing, intracellular ROS levels were quantified by detecting fluorescence with a multimode microplate reader (EnVision, PerkinElmer), and normalized to total protein content. Results are expressed as relative fluorescence units (RFU) per mg protein.

### 2.6 Enzyme-linked immunosorbent assay (ELISA)

To ensure standardized protein concentrations, equal weights of muscle and spinal cord tissue samples from different groups were weighed and homogenized. The concentrations of IL-1β, IL-6, and TNF-*α* in rat serum, muscle, and spinal cord homogenates were then quantified using corresponding ELISA kits (Nanjing Jiancheng Bioengineering Institute). Standard solutions were prepared to generate a calibration curve. Following the kit instructions, samples and reagents were dispensed in sequence. Optical density (OD) was measured at 450 nm utilizing a multimode microplate reader (EnVision, PerkinElmer), and cytokine concentrations were calculated based on the standard curve.

### 2.7 Transcriptome sequencing analysis

Total RNA was extracted from the muscle and spinal cord tissues of rats in the PHN model group and the intra-fascial electrothermal acupuncture (ETA) treatment group using a Trizol kit (TianGen Biochemical Technology Co., Ltd.). After assessing RNA concentration and integrity, qualified samples underwent RNA sequencing with three biological replicates per group.

Raw sequencing reads were processed to generate a gene-level count matrix. Raw sequencing reads were processed to generate a gene-level count matrix. For visualization purposes only, the raw data were log-transformed, quantile-normalized, and batch-adjusted using the ComBat algorithm to generate principal component analysis (PCA) plots. Differential expression analysis between the ETA+PHN and PHN groups was performed using the DESeq2 package (v1.48.1) based on the raw count matrix, with batch included as a covariate in the design formula. Differentially expressed genes (DEGs) were identified using a threshold of *p*-value < 0.05 and |log2(Fold Change)| ≥ 0.5.

### 2.8 Functional enrichment analysis

GO and KEGG enrichment analyses were performed on the candidate genes using the enrichGO and enrichKEGG functions from the clusterProfiler package (v. 4.16.0), with the org. Rn.eg.db database (v3.21.0) serving as the reference. The top 10 significantly enriched terms (p.adjust < 0.05) from each of the GO categories and KEGG pathways were chosen. When p.adjust values were identical, the term with the highest gene count was prioritized. The ggplot2 package (version 3.5.2) was utilized to create the result figures.

### 2.9 PPI network construction and core gene screening

Candidate gene PPI data were sourced from the STRING database^1^. This network was then imported into Cytoscape software (v. 3.10.0) for visualization. Thereafter, core genes were identified using the cytoHubba plugin, which applied 10 algorithmic criteria. The top 10 genes from each algorithm were intersected to determine the final set of core genes.

### 2.10 Regulatory network construction

The miRWalk database^2^ was employed to forecast upstream regulatory miRNAs targeting the core genes. The resulting miRNA-mRNA regulatory relationships were analyzed and visualized as a network using Cytoscape (v. 3.10.0).

### 2.11 RNA extraction and RT-PCR

Rats were anesthetized with 3% sodium pentobarbital (35 mg/kg) and euthanized by cervical dislocation. Muscle and spinal cord tissues from the electroacupuncture intervention site were collected, and total RNA was extracted using TRIzol reagent. One microgram of total RNA was reverse-transcribed into cDNA using a commercial kit (KR116-02, TIANGEN, Beijing, China). Real-time quantitative PCR was performed using SYBR Green PCR master mix (FP205-01, TIANGEN, Beijing, China). Under the following conditions: an initial 30 s at 95 °C, followed by 40 cycles of 5 s at 95 °C, 30 s at 60 °C, and a final extension of 20 min at 72 °C. Following each reaction, melting curve analysis was performed to confirm the absence of primer–dimers. Glyceraldehyde-3-phosphate dehydrogenase (GAPDH) was used as an internal control to normalize variations in RNA quantity and quality among samples. Target mRNA levels were quantified using the 2^ΔΔc(t)^ method. Primer sequences: GAPDH, forward: 5’-GAGTCTACTGGCGTCTTCACC-3′, reverse: 5’-CAGTCTTCTGAGTGGCAGTGAT-3′. MX1, forward: 5’-GCTTCCGTGATGATGCT-3′, reverse: 5’-ACTGAGGCTTGGTTTGCTT-3′. IRF7, forward: 5’-TGCTCCTGGAGCTGGAA-3′, reverse: 5’-GATGTCTCATAGAGGCTGTTGG-3′. Rtp4, forward: 5’-GTGGACCCTGCACTTGGATA-3′, reverse: 5’-TCTGGAACACTGGAACCTGC-3′. oasl2, forward: 5’-ACCTCCGAGGAGCGAG-3′, reverse: 5’-TTCAACTTCCTGATGGGGCT-3′. Ifit1b1, forward: 5’-CTGCCTTGAAATGGAGTGAAGA-3′, reverse: 5’-TGCTTCCAAATCAGGCATGT-3′.

### 2.12 Statistical analysis

Comparisons were made by one-way ANOVA followed by Tukey’s *post hoc* test. A two-way repeated-measures ANOVA was performed to analyze the paw withdrawal threshold (PWT) time-series data, assessing the effects of group, time, and their interaction. *Post hoc* comparisons were performed using Bonferroni correction, and area under the curve (AUC) was calculated via the trapezoidal method, with between-group differences assessed by one-way ANOVA. All statistical tests employed a significance level of *α* = 0.05, and data are presented as mean ± standard deviation (SD). Statistical analyses were carried out using SPSS version 26.0, and graphs were generated using GraphPad Prism version 9.5 (GraphPad Software, California, USA).

## 3 Results

### 3.1 Electrothermal acupuncture alleviates RTX-induced mechanical hypersensitivity

**Figure 1:**
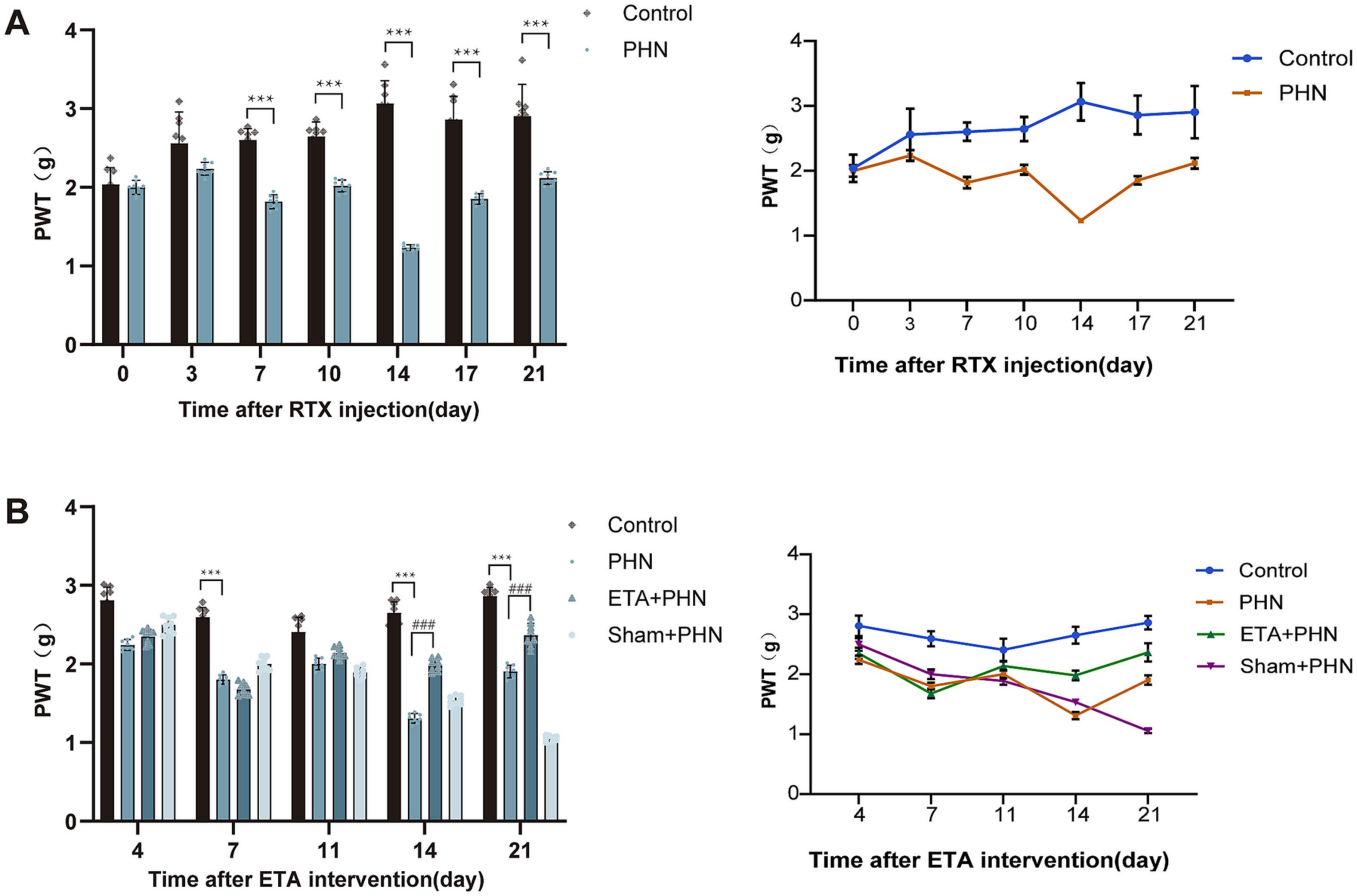
Development of mechanical hyperalgesia in multiple experimental groups. **(A)** Bar and line graphs of intergroup differences and temporal progression of PWT after RTX injection (*n* = 8). **(B)** Bar and line graphs of temporal progression of PWT between groups following ETA (*n* = 8). Data are expressed as mean ± SD. Control: consisted of healthy SD rats that received an intraperitoneal injection of an equivalent volume of physiological saline; PHN: RTX-injected rats (200 μg/kg) to establish the PHN model; ETA + PHN: PHN rats receiving electrothermal acupuncture; Sham + PHN: PHN rats receiving sham procedure. ***p < 0.001 vs. Control; ###*p* < 0.001 vs. PHN group.

Both group (control vs. PHN) and time were found to exert highly significant main effects on PWT (*p* < 0.001). A highly significant group-by-time interaction was also detected (*p* < 0.001), indicating that the effect of RTX modeling on PWT was time-dependent (Table 1). *Post-hoc* multiple comparisons revealed that from day 7 onward, PWT in the PHN group was significantly lower than that in the control group (*p* < 0.001), and this difference persisted through day 21, confirming that RTX modeling successfully induced a neuropathic pain state (Figure 1A and Table 2). No significant difference in area under the curve (AUC) was observed between the two groups, suggesting comparable overall cumulative effects on PWT (Table 3).

**Table 1:** Results of two-way repeated measures ANOVA for the Control and PHN groups.

Source	SS	df	*F*	*p*
Group	16.6883	1	367.0818	<0.001
Time	2.6555	6	9.7352	<0.001
Interaction	7.742	6	28.3828	<0.001
Residual	4.4553	98	–	–

**Table 2:** *Post-hoc* multiple comparisons (Bonferroni-corrected) between the Control and PHN groups yielded the following results.

Time	Control (mean ± SD)	PHN (mean ± SD)	Mean Diff	*p*
0 day	2.039 ± 0.210	1.997 ± 0.090	0.041	0.620175
3 day	2.557 ± 0.400	2.236 ± 0.081	0.322	0.058302
7 day	2.603 ± 0.143	1.818 ± 0.088	0.786	<0.001
10 day	2.646 ± 0.187	2.017 ± 0.076	0.629	<0.001
14 day	3.065 ± 0.289	1.233 ± 0.035	1.832	<0.001
17 day	2.860 ± 0.300	1.853 ± 0.066	1.007	<0.001
21 day	2.904 ± 0.404	2.117 ± 0.082	0.788	<0.001

**Table 3:** AUC quantification analysis results.

Group	*n*	AUC mean ± SD (g·day)	*F*	*p*
Control	8	7.191 ± 4.691	1.271	0.279
PHN	8	4.921 ± 3.232	–	–

As shown in Table 4, the two-way ANOVA revealed significant main effects of both group (*F* = 97.636, *p* < 0.001) and time (*F* = 83.243, p < 0.001), as well as a significant group-by-time interaction (*F* = 94.737, p < 0.001). *Post hoc* comparisons showed that from day 7 to day 21, PWT in the control group was significantly higher than that in all other groups (all p < 0.001). Moreover, PWT in the ETA + PHN group was significantly elevated compared to the PHN group alone (*p* < 0.001, Figure 1B, Table 5). As shown in Table 6, AUC analysis demonstrated that the control group had the highest overall PWT (44.565 g/day), while the cumulative effect in the ETA + PHN group (35.064 g/day) was significantly greater than that in the PHN group and the sham surgery + PHN group (28.733 g/day). These results indicate that ETA significantly attenuates mechanical pain sensitivity in PHN rats in a time-dependent manner. Moreover, AUC values differed significantly across the four groups (*p* < 0.001), reflecting a statistically significant effect of the interventions on pain thresholds in PHN rats.

**Table 4:** Results of two-way repeated measures ANOVA for the four groups.

Source	SS	df	*F*	*p*
Group	5.7095	3	97.6358	<0.001
Time	6.4904	4	83.2428	<0.001
Interaction	22.1598	12	94.737	<0.001
Residual	2.7484	141	–	–

**Table 5:** *Post-hoc* multiple comparisons (Bonferroni-corrected) between the four groups yielded the following results.

Time	Group	Mean Diff	p
7 day	Control vs. PHN	0.791	<0.001
7 day	ETA + PHN vs. PHN	−0.123	0.0036
14 day	Control vs. PHN	1.341	<0.001
14 day	ETA+PHN vs. PHN	−0.670	<0.001
21 day	Control vs. PHN	0.957	<0.001
21 day	ETA + PHN vs. PHN	−0.461	<0.001

**Table 6:** AUC quantification analysis results.

Group	*n*	AUC mean ± SD (g·day)	*F*	*p*
Control	8	10.8218 ± 0.4527	253.4058	<0.001
PHN	8	6.9697 ± 0.2512	–	–
Sham + PHN	8	7.0324 ± 0.2353	–	–
ETA + PHN	8	8.6039 ± 0.2995	–	–

Data are presented as mean ± SD indicates sample size per group. Control: healthy Sprague–Dawley rats; PHN: rats receiving RTX injection; ETA + PHN: PHN rats receiving daily 15-min electrothermal acupuncture (42 °C) at 6 mm depth on hyperalgesic sites; Sham + PHN: PHN rats undergoing identical procedure with needle placement on skin surface without insertion.

### 3.2 Electrothermal acupuncture modulates mitochondrial function and inflammatory responses

**Figure 2:**
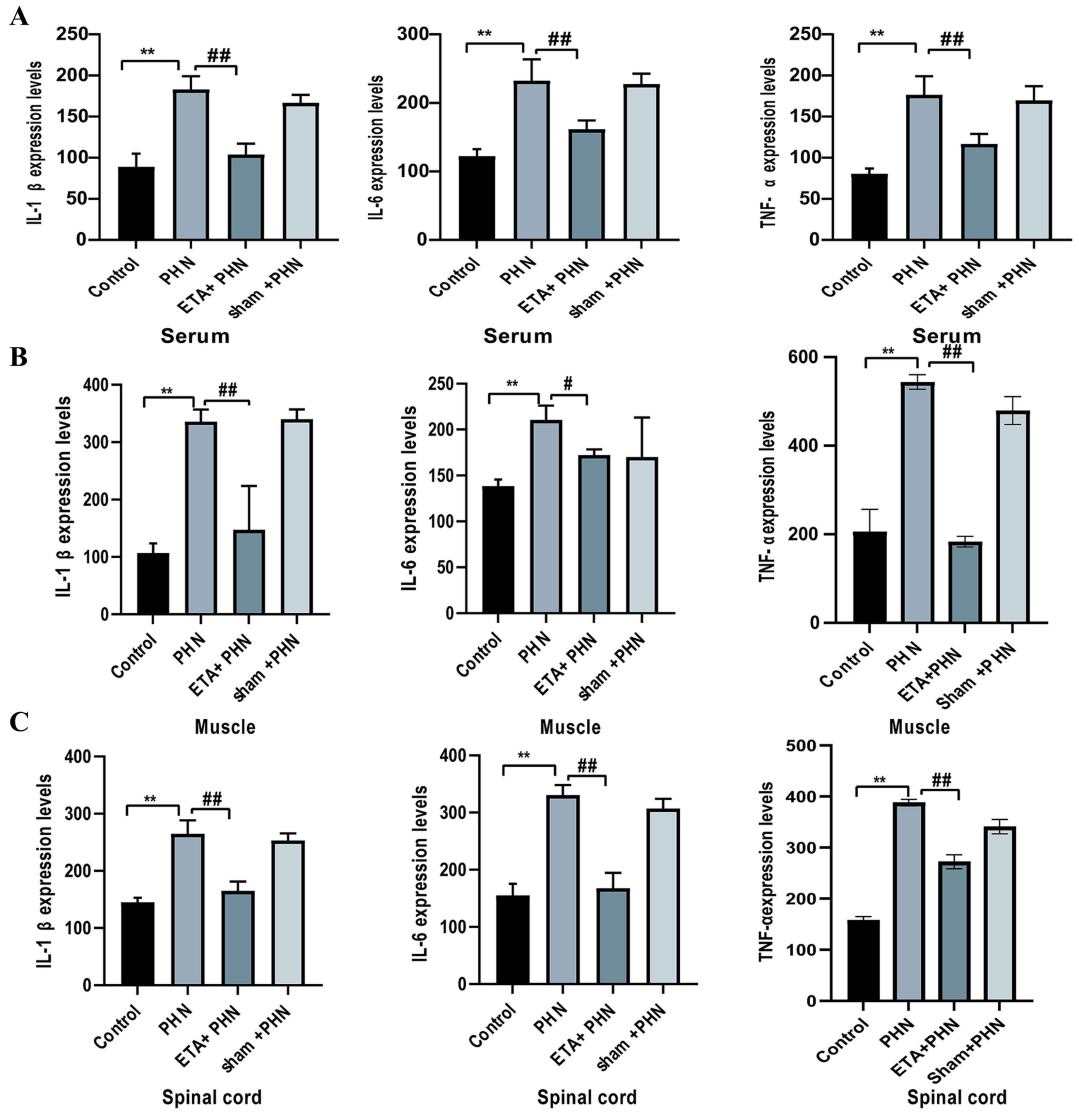
ETA improves modulates inflammatory response. **(A)** The IL-1β, IL-6, and TNF-*α* levels were measured in rat serum by ELISA; **(B)** the IL-1β, IL-6, and TNF-α levels were measured in rat muscle tissue homogenates by ELISA; **(C)** the IL-1β, IL-6, and TNF-α levels were measured in rat spinal cord tissues homogenate by ELISA. To eliminate errors caused by variations in tissue sample weights, the measured concentration of each cytokine (pg/mL) obtained from the ELISA was divided by the total protein concentration (mg/mL) of the corresponding tissue homogenate. The final data are presented as the cytokine content per milligram of protein (pg/mg protein). Data are expressed as mean ± SD (*n* = 6 per group). *p* < 0.01 vs. Control; #*p* < 0.05, ##*p* < 0.01 vs. PHN group (one-way ANOVA with Turkey’s *post hoc* test).

To further evaluate the therapeutic effects of ETA, we measured the expression levels of pro-inflammatory cytokines (IL-1β, IL-6, and TNF-*α*) in rat serum, muscle, and spinal cord tissues. The results are presented in Table 7 and Figure 2. Compared with the Control group, levels of all three pro-inflammatory cytokines were significantly elevated (*p* < 0.01) in the serum, spinal cord, and muscle tissues of rats in the PHN model group, indicating that the neuropathic pain model successfully induced both systemic and localized inflammatory responses. The anti-inflammatory effect of ETA intervention was consistently observed across all examined tissues: In serum, ETA treatment significantly attenuated the PHN-induced increases in IL-1β, IL-6, and TNF-*α* levels (*p* < 0.05 or *p* < 0.01), reducing their expression to levels intermediate between the Control and PHN model groups. In the spinal cord, ETA effectively reversed the aberrant overexpression of IL-1β, IL-6, and TNF-α (*p* < 0.01), with a particularly pronounced inhibitory effect on TNF-α. In muscle tissue (the peripheral site of pain), ETA demonstrated a potent local anti-inflammatory effect. Following treatment, the levels of all three inflammatory cytokines were significantly suppressed (*p* < 0.01). Notably, the levels of IL-6 and TNF-α after ETA treatment were restored to levels comparable to or even lower than those in the Sham + PHN group. Furthermore, the levels of each inflammatory cytokine in the Sham + PHN group were significantly higher than those in the Control group but lower than those in the PHN model group, which aligns with expectations and further confirms the reliability of the specific inflammatory response in the PHN model. In conclusion, these results demonstrate that ETA can effectively inhibit the central and peripheral inflammatory responses induced by PHN, which may represent one of the key mechanisms underlying its efficacy in alleviating neuropathic pain.

**Table 7:** Effects of intrafascial electrothermal acupuncture on inflammatory factors (x ± s, *n* = 6, μg/L).

Sample source	Name	Control	PHN (*n* = 6)	ETA + PHN (*n* = 6)	Sham + PHN (*n* = 6)
Serum	IL-1β	88.58 ± 11.72	182.91 ± 16.3	103.57 ± 13.71	166.5 ± 10.14
	IL-6	121.94 ± 10.34	232.21 ± 31.37	161.3 ± 13.13	227.41 ± 15.13
	TNF-α	80.37 ± 6.48	176.28 ± 22.97	116.53 ± 12.47	169.42 ± 17.62
Spinal cord	IL-1β	145.31 ± 7.91	264.84 ± 23.76	165.06 ± 16.34	253.27 ± 12.39
	IL-6	155.24 ± 20.35	330.54 ± 17.53	167.64 ± 26.7	307.27 ± 16.89
	TNF-α	158.32 ± 6.67	387.92 ± 6.3	272.5 ± 13.86	341.16 ± 14.09
Muscle	IL-1β	106.82 ± 16.7	335.67 ± 21.3	147.39 ± 9.23	340.19 ± 17.01
	IL-6	138.47 ± 7.03	270.43 ± 15.8	130.8 ± 6.24	170.2 ± 43.1
	TNF-α	206.15 ± 50.3	543.8 ± 16.4	183.61 ± 11.8	479.34 ± 31.5

**Figure 3:**
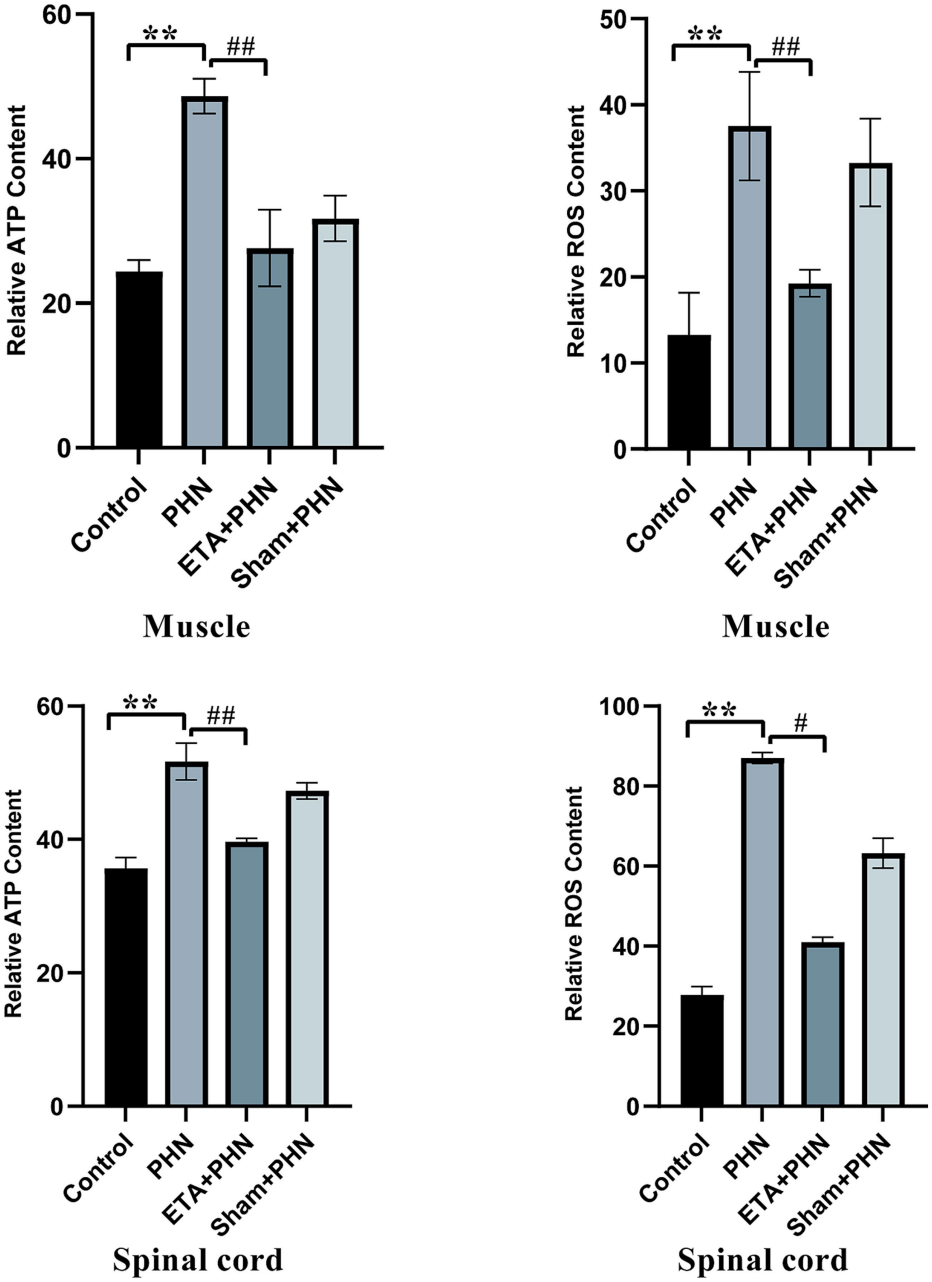
ETA improves ROS and ATP. Protein concentration was determined by nano spectrophotometer assay to ATP and ROS levels, the raw data were normalized to the total protein concentration of each sample (expressed as /mg protein). Data are expressed as mean ± SD (*n* = 6 per group). *p* < 0.01 vs. Control; #*p* < 0.05, ##*p* < 0.01 vs. PHN group (one-way ANOVA with Turkey’s post hoc test).

To explore the potential mechanism underlying the anti-inflammatory and analgesic effects of ETA, we assessed the levels of ATP and ROS in muscle and spinal cord tissues. As shown in Table 8 and Figure 3, compared to the Control group, the levels of both ATP and ROS were significantly elevated (*p* < 0.01) in the muscle and spinal cord tissues of rats in the PHN model group. Furthermore, ETA treatment significantly reversed the PHN-induced excessive increases in ATP and the abnormal accumulation of ROS in both tissues (*p* < 0.01). Notably, in muscle tissue, ETA restored the levels of ATP and ROS to a range comparable to that of the Sham + PHN group. In the spinal cord, the inhibitory effect of ETA on ROS was particularly pronounced, reducing its level to significantly below that of the Sham + PHN group. These results indicate that ETA can simultaneously modulate energy metabolism and redox status in pain-related tissues, and its effective reduction of ROS may be a key link in exerting its anti-inflammatory and analgesic effects.

**Table 8:** Effects of intrafascial ETA on ATP and ROS (x ± s, *n* = 6, μg/L).

Sample source	Name	Control	PHN (*n* = 6)	ETA + PHN (*n* = 6)	Sham + PHN (*n* = 6)
Muscle	ATP	24.37 ± 1.6	48.67 ± 2.4	27.63 ± 5.3	31.71 ± 3.16
	ROS	13.28 ± 4. 9	37.54 ± 6.31	19.27 ± 1.58	33.29 ± 5.1
Spinal cord	ATP	35.64 ± 1.67	51.67 ± 2.75	39.67 ± 0.47	47.3 ± 1.24
	ROS	27.81 ± 2.1	87.03 ± 1.37	41.05 ± 1.16	63.24 ± 3.74

Data are presented as mean ± SD. n indicates sample size per group. Control: healthy Sprague–Dawley rats; PHN: rats receiving RTX injection; ETA+PHN: PHN rats receiving daily 15-min electrothermal acupuncture (42 °C) at 6 mm depth on hyperalgesic sites; Sham + PHN: PHN rats undergoing identical procedure with needle placement on skin surface without insertion.

### 3.3 Screening of differentially expressed genes

**Figure 4:**
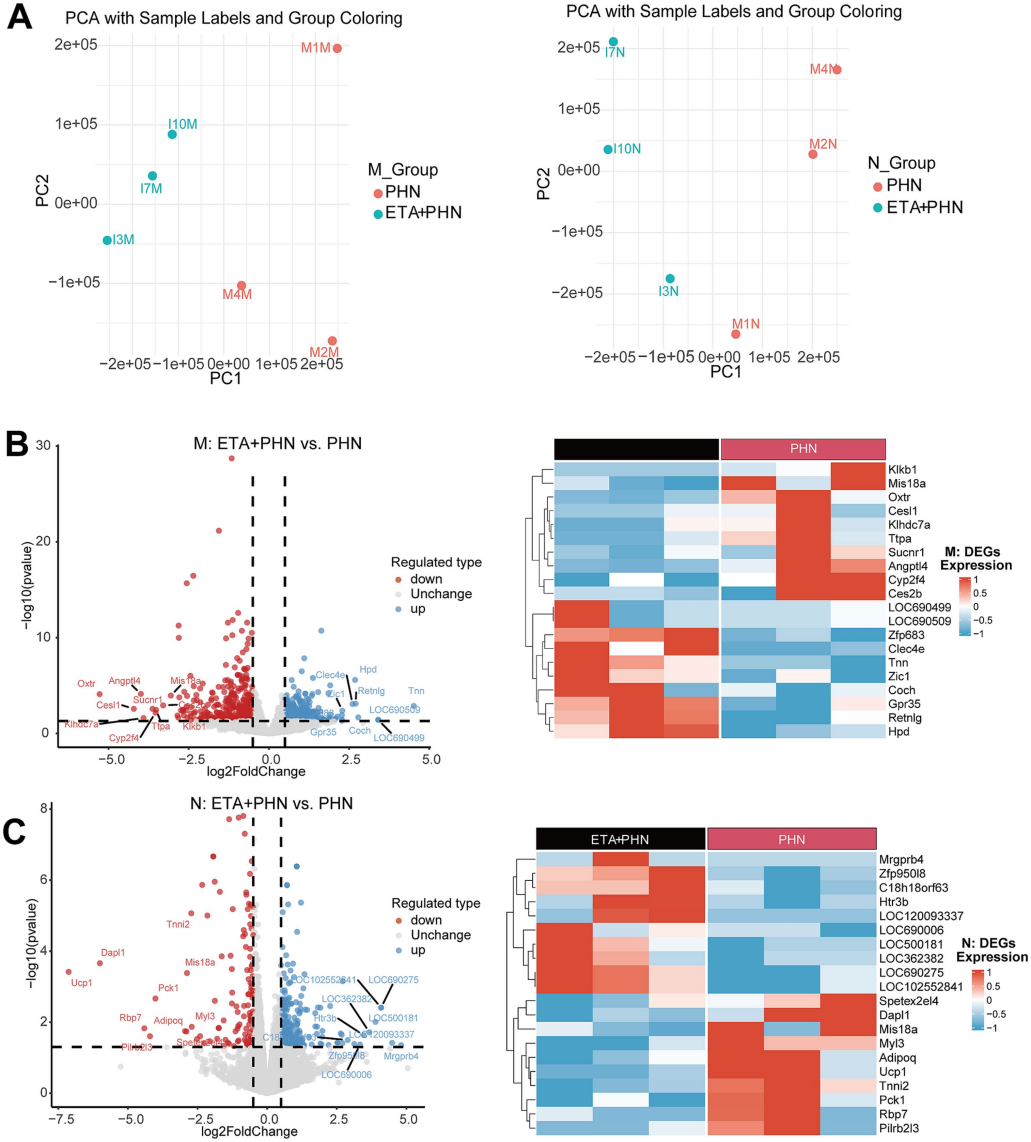
Transcriptomic profiling reveals tissue-specific gene expression changes. **(A)** The PCA plots depict transcriptional variation in muscle (left panel) and spinal cord (right panel) tissue samples from the PHN and ETA+PHN groups. Each point represents an individual sample, with color indicating group assignment (red: PHN; cyan: ETA + PHN). Sample names are labeled. The first and second principal components (PC1 and PC2, horizontal and vertical axes respectively) correspond to the two largest sources of variation. **(B)** Analysis of muscle tissue. **(C)** Analysis of spinal cord tissue. For both **(B,C)**, the left panel shows a volcano plot of DEGs between the PHN and ETA + PHN groups. Significantly upregulated genes are denoted by red points, downregulated genes by blue points, and non-significant genes by gray points. The horizontal dashed line indicates the threshold for statistical significance (*p*-value < 0.05), and the vertical dashed lines mark a |log2FC| of 0.5. The right panel presents a clustered heatmap of the significant DEGs (|log2FC| ≥ 0.5 and *p*-value < 0.05), where red and blue again represent upregulated and downregulated expression, respectively. M: Muscle tissue. N: Spinal cord tissue.

To elucidate the molecular mechanisms of ETA, mRNA sequencing was performed on muscle and spinal cord tissues from PHN and ETA groups. Principal Component Analysis (PCA) demonstrated clear separation between the PHN and ETA+PHN groups in both muscle and spinal cord tissues (Figure 4A). In muscle tissue, 822 differentially expressed genes (DEGs) were identified (476 downregulated, 346 upregulated, Figure 4B). Spinal cord tissue yielded 333 DEGs (220 upregulated, 113 downregulated, Figure 4C). The screening threshold was |log2(FC)| ≥ 0.5 with *p*-value < 0.05. The specific list of DEGs is provided in Supplementary Table S1.

### 3.4 Functional enrichment of candidate genes

**Figure 5:**
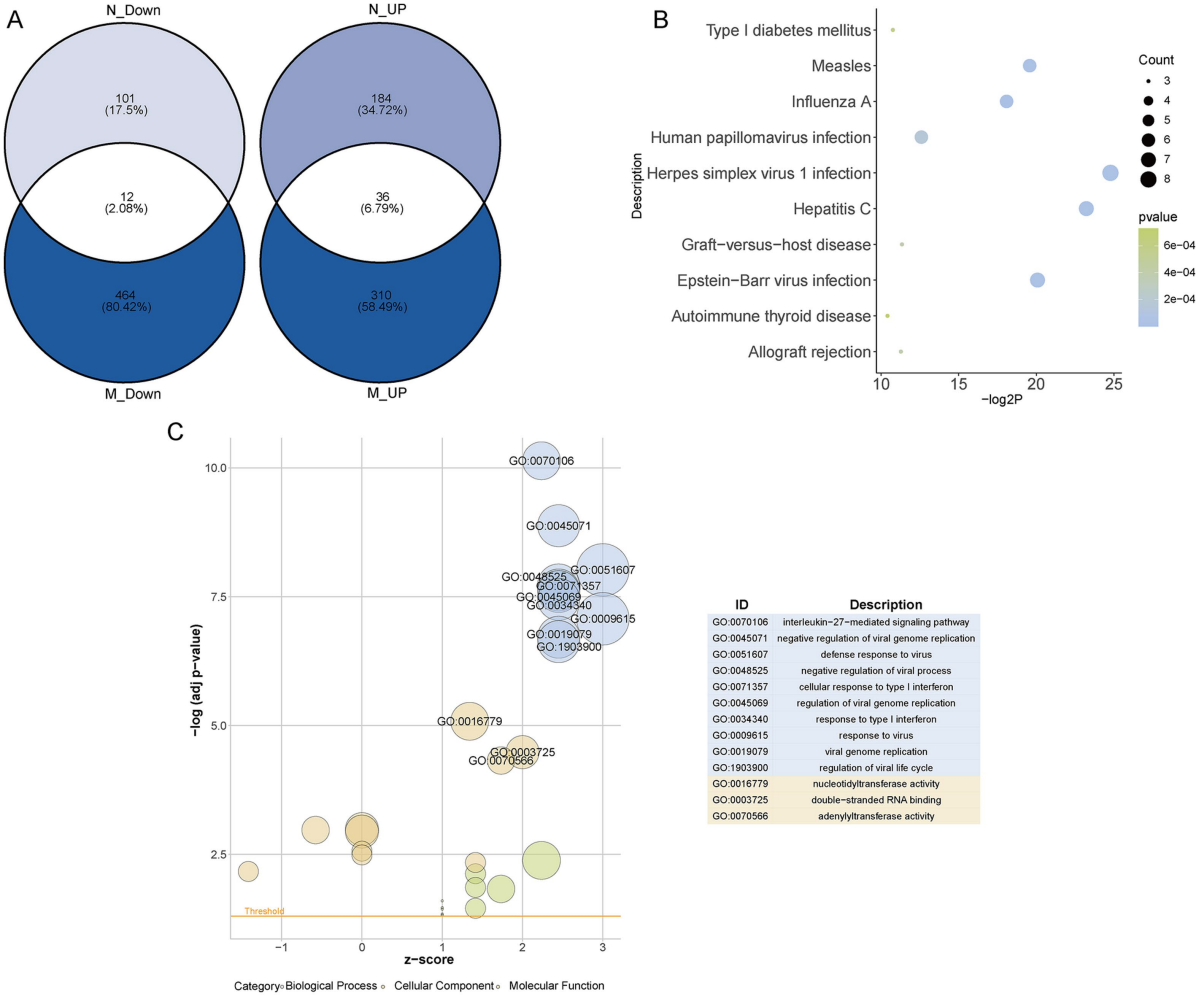
Functional enrichment analysis of candidate genes. **(A)** Venn diagrams depict the overlap of up- and down-regulated differentially expressed genes (DEGs) between muscle and spinal cord tissues, identifying 48 shared DEGs. **(B)** KEGG pathway enrichment analysis of candidate genes. **(C)** GO functional enrichment analysis of candidate genes. Bubble size represents gene count; color intensity indicates statistical significance.

A Venn diagram analysis identified 48 DEGs common to both muscle and spinal cord tissues (Figure 5A). Specifically, 12 downregulated DEGs originated from the intersection of the 476 downregulated DEGs in muscle and the 113 downregulated DEGs in spinal cord. The remaining 36 common DEGs were upregulated, derived from the intersection between the 346 upregulated DEGs in muscle and the 220 upregulated DEGs in spinal cord. The specific list of DEGs is provided in Supplementary Table S1. The 48 DEG defined as candidate genes for ETA action. KEGG pathway analysis revealed significant enrichment of the core genes in several pathways annotated with viral infections (e.g., Human papillomavirus infection, Herpes simplex virus 1 infection). Notably, these pathways are known to encompass a robust set of interferon-stimulated genes (ISGs) and shared host defense modules. Therefore, this enrichment pattern likely reflects the activation of a broad type I interferon-mediated innate immune response and inflammatory signaling, rather than implicating specific viral activity in our model (Figure 5B). According to GO analysis, these genes are primarily involved in type I interferon signaling and related inflammatory processes (e.g., interleukin-27-mediated signaling pathway, cellular response to type I interferon, response to type I interferon, and the virus-responsive host-defense module). This enrichment pattern indicates that the genes function within a broad innate immune and inflammatory program activated by RTX, rather than denoting a specific antiviral state (Figure 5C).

### 3.5 Identification of core genes and construction of regulatory networks

**Figure 6:**
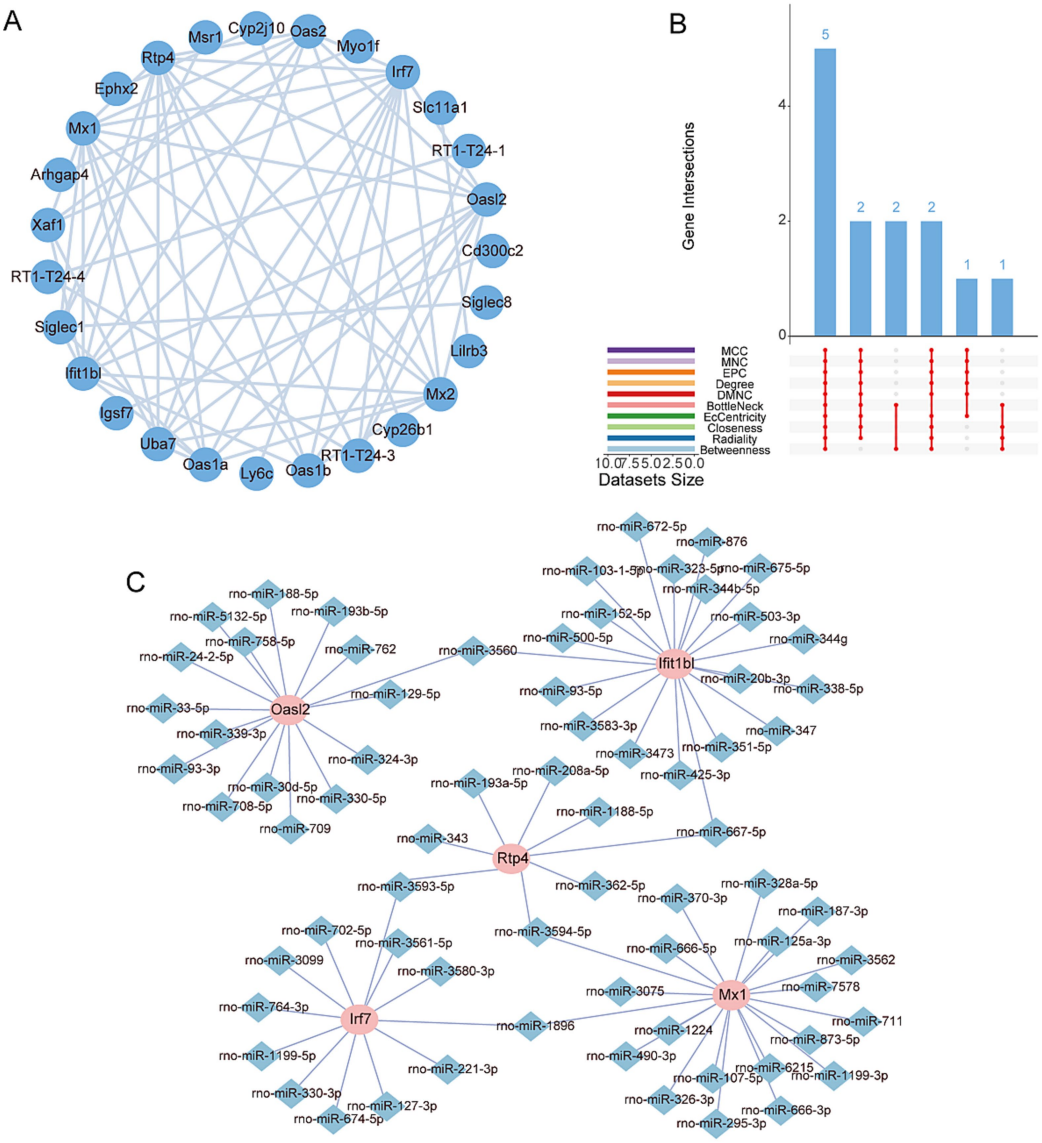
Screening of core genes and construction of regulatory networks. **(A)** Protein–protein interaction (PPI) network of candidate genes. **(B)** Bar graph showing intersection counts of top-ranked genes identified by different CytoHubba algorithms. **(C)** miRNA-mRNA regulatory network depicting shared miRNAs targeting core genes.

A PPI network was constructed from the candidate genes using data from the STRING database and visualized in Cytoscape (Figure 6A). Cross-analysis using multiple algorithms in CytoHubba identified five core genes: Mx1, Irf7, Rtp4, Oasl2, and Ifit1bl (Figure 6B). The miRWalk database was used to predict upstream miRNAs for these core genes, and a regulatory network was built. As shown in Figure 5C, rno-miR-3593-5p co-regulating Irf7 and Rtp4; rno-miR-1896 co-regulates Irf7 and Mx1; rno-miR-3560 co-regulating Oasl2 and Ifit1bl; rno-miR-667-5p co-regulating Rtp4 and Ifit1bl; and rno-miR-3594-5p co-regulating Irf7 and Mx1.

### 3.6 Expression of core genes in rat spinal cord and muscle tissue

**Figure 7:**
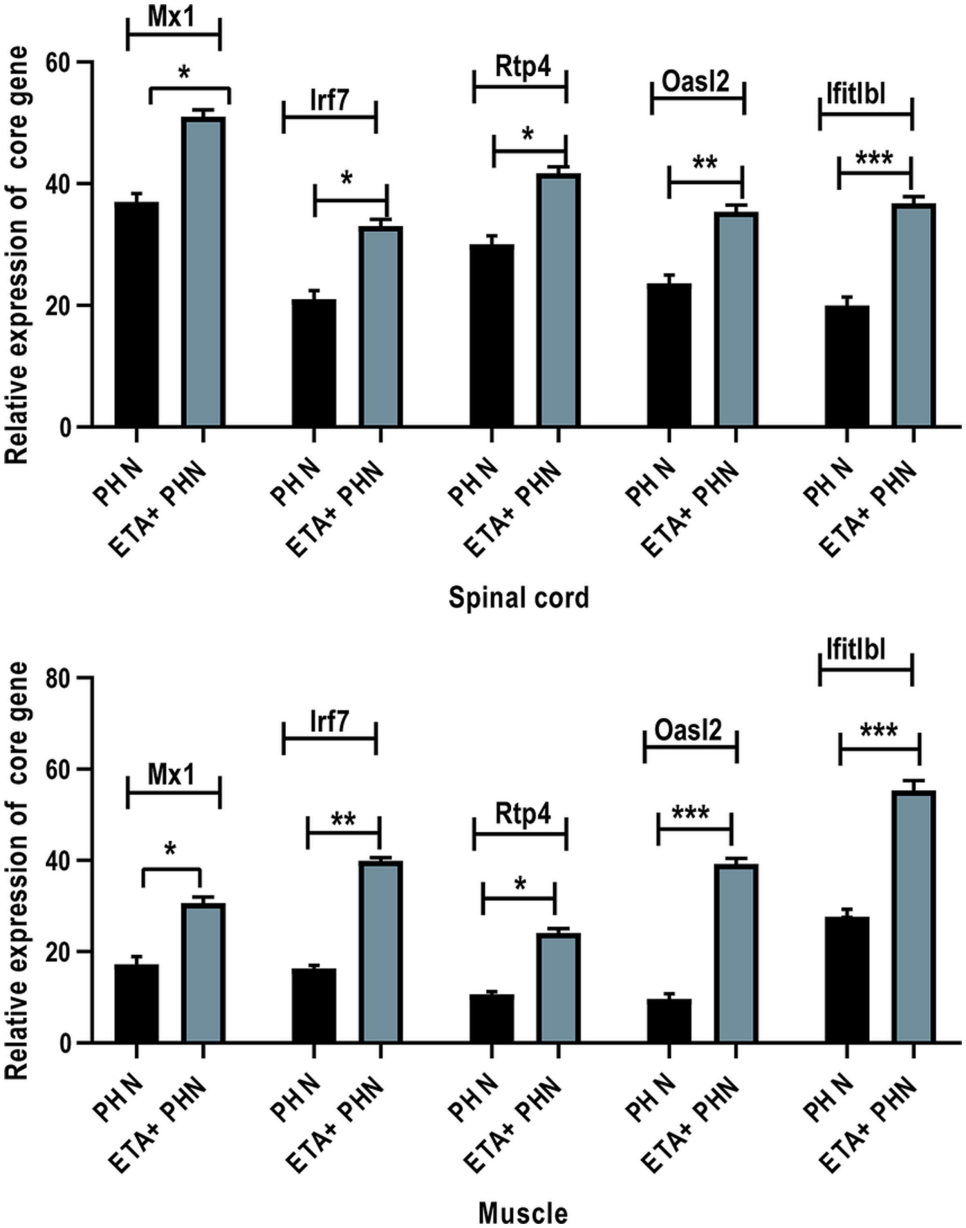
Relative mRNA expression levels of core genes (*Mx1*, *Irf7*, *Rtp4*, *Oasl2*, *Ifit1bl*) in muscle and spinal cord. Glyceraldehyde-3-phosphate dehydrogenase (GAPDH) was used as an internal control. Data are presented as mean ± SD from three independent experiments, each performed in triplicate. Statistical significance was determined using one-way ANOVA followed by Turnkey’s post hoc test.

To validate the RNA-seq findings, the expression levels of five core genes (Mx1, Irf7, Rtp4, Oasl2, and Ifit1bl) were quantified. Consistent with the sequencing data, qRT-PCR analysis confirmed that the mRNA expression levels of Mx1, Irf7, Rtp4, Oasl2, and Ifit1bl were significantly upregulated in the ETA+PHN group compared to the PHN group (*p* < 0.05; Figure 7).

## 4 Discussion

PHN is a prevalent and refractory neuropathic pain condition that severely impairs quality of life. ETA, which integrates traditional acupuncture with electrical and thermal stimulation, has emerged as a promising therapy. It enhances local metabolism and controls inflammation, among other benefits (21). Consistent with previous studies and the documented neuromodulatory effects of electroacupuncture (13, 22, 23), our results demonstrate that ETA significantly elevates the PWT in PHN rats, confirming its analgesic effect. Research suggests that electroacupuncture can improve cellular energy metabolism by modulating mitochondrial function, promoting ATP synthesis, and consequently reducing reduced oxidative stress (ROS) production to alleviate oxidative stress and inflammation (24). Aligning with this, our study found that ETA reversed the RXT-induced excessive ATP elevation and abnormal ROS accumulation in both tissues (*p* < 0.01), with ETA intervention showing particularly marked suppression of ROS in spinal cord tissue. This aligns with findings in denervation-induced atrophy, where therapeutic intervention simultaneously ROS and improved tissue outcomes (25). ETA treatment significantly attenuated RXT-induced increases in the levels of IL-1β, IL-6, and TNF-*α* (*p* < 0.05). Many studies report that electroacupuncture significantly lowers levels of IL-6, IL-1β, and TNF-α, reduces inflammatory cell infiltration, and inhibits neuronal apoptosis (24, 26, 27). This anti-inflammatory effect is largely attributed to the inhibition of the nuclear factor kappa-B (NF-κB) signaling pathway. NF-κB is a master transcriptional regulator of inflammation, and its activation directly promotes the transcription of TNF-α, IL-1β, and IL-6 (28). Consistent with this, prior evidence demonstrates that electroacupuncture can downregulate NF-κB p65 expression and ameliorate microglial-mediated inflammation (29). The anti-inflammatory effect of acupuncture modalities is further supported by evidence showing reduced activation of macrophages/microglia (marked by CD68) in injured nervous tissue following treatment (13). To gain deeper mechanistic insights, we conducted transcriptome sequencing, identifying 48 common DEGs in muscle and spinal cord tissues. These genes were predominantly enriched in pathways involving interferon-mediated innate immune-related pathways. The prominence of interferon-related pathways is particularly interesting. As interferon signaling is a key component of the neuroimmune response and has been implicated in the glial cell dialogue that sustains neuroinflammation in chronic pain conditions (9). However, since these findings are derived from transcriptomic pathway enrichment analysis, further experimental validation is warranted to ascertain that the observed enrichment of viral infection-associated pathways reflects bona fide activation of type I interferon-mediated innate immune signaling, rather than any direct viral involvement within the RTX-induced model. Subsequent PPI network analysis predicts five core genes: Mx1, Irf7, Rtp4, Oasl2, and Ifit1bl.

Mx1, a GTPase induced by interferons, confers resistance to various viruses (30). Its expression correlates positively with antioxidant enzyme activity (31) and is regulated by mitochondrial ROS, which can enhance interferon signaling and type I interferon-stimulated genes (ISGs) expression (32). Conversely, Mx1 expression is negatively correlated with inflammatory cytokines (33), which are known promoters of neuropathic pain (34–36). Upregulation of Mx1 in CD4 + T cells is linked to enhanced mitochondrial activity and energy metabolism (37), while its high expression may reflect cellular stress in neuroinflammation (38). Irf7 initiates the expression of type I interferons (IFNα/*β*), thereby activating downstream innate immune response (39). Beyond playing a role in the type I interferon-mediated innate immune response, Irf7 is implicated in oxidative stress by activating the Nrf2 pathway (40). Additionally, it promotes inflammatory gene transcription, which contributes to inflammation (41, 42). Furthermore, Irf7 is also associated with mitochondrial dysfunction and senescence (43). Rtp4 is involved in receptor transport and stability. Its expression in microglia is upregulated by LPS-induced inflammation via TLR4 and JAK–STAT pathways, suggesting a role in neuroinflammation (44). Oasl2, functionally similar to human OASL, is interferon-induced and contributes to type I interferon-mediated innate immune response (45). It can also suppress inflammation by inhibiting the cGAS-STING pathway during DNA viral infection (46). Ifit1bl, a member of the interferon-induced protein family, is upregulated upon interferon pathway activation, which may participate in initiating neuroinflammation (47). Previous studies have shown that RTX suppresses the skin injury-induced type I interferon-mediated innate immune response by disrupting the TRPV1 pathway, downregulating type I interferon signalling pathway-related Proteins such as Oas2, Oasl2, and Mx1 (48). In line with this, our study showed that RTX injection significantly reduced the expression of Mx1, Irf7, Rtp4, Oasl2, and Ifit1bl. Conversely, ETA treatment upregulates these interferon-related genes and alleviates RTX-induced tactile allodynia. While the suppression of Type I interferon signaling by RTX via TRPV1 desensitization is established, the precise mechanism by which ETA restores the expression of these ISGs requires further mechanistic speculation. Based on the current data and the interconnected nature of oxidative stress, inflammation, and interferon responses, we propose two non-mutually exclusive hypotheses. First, the NF-κB-IRF axis crosstalk. As noted above, ETA significantly inhibited the overproduction of IL-1β and TNF-*α*. Given that NF-κB activation is known to compete with or inhibit certain interferon regulatory factor (IRF)-mediated transcriptional programs, the suppression of NF-κB activity by ETA may relieve a transcriptional “brake” on ISG expression. This creates a permissive cellular environment for baseline or residual interferon signaling, thereby allowing the recovery of Mx1 and Irf7 levels. Second, the ROS-mediated mitochondrial retrograde signaling. RTX-induced ATP depletion and ROS accumulation can trigger mitochondrial dysfunction, which has been shown to dampen the cell’s capacity to mount an effective interferon response. By reversing excessive ROS accumulation, ETA may restore mitochondrial homeostasis. This restoration could re-sensitize the cGAS-STING or RIG-I-like receptor pathways to endogenous damage signals, thereby facilitating the upregulation of Oasl2 and Ifit1bl independent of viral infection. This hypothesis aligns with the concept that therapeutic reduction of oxidative stress correlates with improved ISG expression profiles (31, 32). The observed protective effects of ETA resonate with broader findings regarding neuromodulation and pain relief. Notably, Xu et al. (49) demonstrated that electroacupuncture attenuates cancer-induced bone pain specifically by inhibiting the NF-κB/CXCL12 signaling axis in the periaqueductal gray, a mechanism that restrains descending pain facilitation. Furthermore, Zhao et al. (50) showed that catalpol ameliorates inflammatory pain by targeting both spinal cord and peripheral inflammation. Parallel to these studies, our findings show that ETA may similarly engages a dual mechanism: (1) peripheral and spinal suppression of NF-κB-mediated inflammatory cascades (evidenced by reduced IL-1β/IL-6), and (2) restoration of the interferon-mediated innate immune/neuroprotective signaling (evidenced by upregulation of Mx1/Irf7). Our data extend this by revealing a potential intrinsic cellular repair mechanism involving ISGs in the affected tissues. Considering these findings, the analgesic effect of ETA in PHN appears to stem from a coordinated resolution of neuroinflammation and a restoration of neuroimmune homeostasis.

This study has several limitations. First, the miRNA–mRNA regulatory network predicted using the miRWalk database represents a computational inference; therefore, independent validation through *in vivo* animal studies or *in vitro* cellular models is required to substantiate these predicted. Second, the interactions between pathways related to these core genes and their upstream/downstream signaling cascades require further elucidation. Third, potential tissue-specific effects of ETA (e.g., differential responses in muscle versus spinal cord) need more detailed investigation. Fourth, the ISGs identified herein (Mx1, Irf7, Rtp4, Oasl2, and Ifit1bl) represent hypothesis-generating observations, underscoring the need for further animal studies utilizing gene interference or pathway inhibition to establish causal relationships. Employing gene editing techniques, cellular models, and comprehensive behavioral assays in future studies will be crucial to systematically validate the predictive role and functional significance of these core genes in ETA treatment for PHN. While the RTX-induced model effectively replicates the thermal hypoalgesia with mechanical allodynia of clinical PHN, the RTX model bypasses the critical phase of viral reactivation and latency in the dorsal root ganglia. Therefore, the therapeutic effects of ETA observed in this study are primarily reflective of its capacity to modulate established neuropathic pain, rather than its antiviral or acute anti-herpetic effects. Future studies may benefit from combining these findings with HSV-1 models to fully elucidate the spectrum of ETA’s therapeutic actions. Furthermore, future studies will incorporate animal behavioural indicators of thermal allodynia and spontaneous pain to provide a more comprehensive characterization of PHN-like phenotypic features.

## 5 Conclusion

In conclusion, electrothermal acupuncture treatment at specified parameters (6 mm insertion, 42 °C, 15 min) effectively mitigated RTX-induced tactile allodynia in a rat model. Mechanistically, the treatment upregulated a set of type I interferon pathway genes, including Mx1, Irf7, Rtp4, Oasl2, and Ifit1bl. The observed changes in gene expression are associated with decreased cellular oxidative stress and reduced inflammation, suggesting that mitigation of inflammation and oxidative stress represents a key mechanism underlying the analgesic effect. These results provide new insights into the molecular basis of electrotherapy, contributing significantly to the understanding of its application in PHN management.

## Data Availability

The original contributions presented in the study are included in the article/Supplementary material, further inquiries can be directed to the corresponding author/s.

## Footnotes

1
https://string-db.org/

2
http://mirwalk.umm.uni-heidelberg.de/
