# Supplementary material for: Mechanism of electrothermal acupuncture in alleviating postherpetic neuralgia
Source: Front Neurol. 2026 Jun 8;17:1750292. doi: 10.3389/fneur.2026.1750292 (PMC13283803; doi:10.3389/fneur.2026.1750292)
Supplement: Supplementary file 1 [file Data_Sheet_1.PDF]

DEGs in muscle tissue_0.5

gene	log2FoldChange
Ucp1	-7.128634091
Dapl1	-6.006193155
Rbp7	-4.411611277
Pilrb2l3	-4.207145568
Pck1	-4.013395917
Adipoq	-2.950075133
Spetex2el4	-2.897703128
Mis18a	-2.879149868
Tnni2	-2.724759287
Myl3	-2.70824933
Xirp2	-2.519280917
Fabp4	-2.407198408
Ckm	-2.333473341
LOC1025481	-2.263812324
Myl1	-2.146467492
Rpph1	-2.057827857
Myh1	-1.935622147
Myh4	-1.935622147
Atp2a1	-1.883379831
Nkx2-8	-1.879814256
Marveld3	-1.795638905
Rpl10al3	-1.74677508
Myoz1	-1.729705796
Tnnt3	-1.69813318
Postn	-1.631475172
Myh2	-1.563051229
Ptgdrl1	-1.557008116
Apold1	-1.497475429
Polq	-1.412282036
Sag	-1.367357916
Acta1	-1.365208288
Clca1	-1.315633799
Cidea	-1.26761523
Ttn	-1.265179329
Cldn14	-1.256995401
Capn6	-1.235599225
Eln	-1.192944159
Cryba4	-1.137459671
RT1-Ba	-1.10379712
Gpx2	-1.065846601
Dmrtb1	-1.05894759
Trim55	-1.054740518
Casr	-1.042056559
Cdkn1c	-1.023972199
Tdrd12	-1.023935024
Actn3	-0.990069209
Cdkn1a	-0.861143033
Gpr17	-0.856495356

Cyp2j10	-0.841041284
Bmp4	-0.832906135
Depdc1b	-0.828118799
Atp1a4	-0.809405931
Sema4d	-0.804675963
Pnlip	-0.797726662
C5h1orf21(	-0.79153851
Rpe65	-0.762514841
Myl11	-0.758295134
Spag5	-0.751669858
Ncaph	-0.750669197
Dynlt1	-0.747976898
Umodl1	-0.735569718
Sema5b	-0.71901372
Rassf10	-0.705770675
Acot1	-0.703990217
Nkd1	-0.699405567
Ephx2	-0.686751889
Kcns1	-0.683008763
Spetex2el1	-0.675099749
Spetex2l2	-0.675099749
Enpp6	-0.668382989
Zfp658l1	-0.665141304
Ginm1	-0.655092605
Rgs22	-0.64544016
Tmem125	-0.625014292
Nfe2l3	-0.623799527
Ddc	-0.618188832
Pex11a	-0.615382407
Mylk	-0.61449528
Scd	-0.61339132
Gal3st1	-0.611830391
Rab37	-0.608942247
Ccdc77	-0.603758206
Cdsn	-0.6026576
Rnf122	-0.600279362
LOC1036899	-0.59465034
Pxmp4	-0.586641883
Mal1	-0.586447123
Abhd15	-0.585370163
Tmem14cl1	-0.581971917
Marcksl1	-0.581482762
Pnpla3	-0.573558497
Thsd7b	-0.573392349
Mycl	-0.570923207
Cfap20dc	-0.563991783
Prr5l	-0.555774616
Ugt8	-0.554817872
Tm7sf2	-0.54022317
Neu4	-0.539852046
Spon2	-0.5359687

Itpr2	-0.529073998
Fam83d	-0.527222279
Rab7b	-0.52701382
Thrsp	-0.525152469
Tmem82	-0.524484641
Spetex2el5	-0.52294049
Id1	-0.51923039
C1ql1	-0.516966216
Tmem117	-0.512736529
Fa2h	-0.510278829
Nipal4	-0.507700114
Ramp3	-0.503898519
Chst4	-0.502673574
Mbp	-0.502073555
Cfap44	0.880529985
Fastkd5	0.501187912123077
F3	0.501394173464107
Chi3l1	0.506501806647281
Catip	0.507320214938535
Ttc14	0.508377185473483
Nat8b	0.50865689094396
Arhgap4	0.510048822300019
Dtx3l	0.515196059459054
Lpar6	0.515348170679108
Invs	0.516028018488273
Rnpc3	0.516477788363954
Cacnb4	0.517729575718289
LOC12010280	0.517998652559662
Krt2	0.522538851976956
Leng8	0.522697457636167
Bod1l1	0.522820076045518
Psmb9	0.524071859643123
Six4	0.524524041586062
Pyroxd2	0.52499162296003
Zfp945	0.527957337999903
Parp9	0.528678189301789
Zfp347	0.529329025004921
Lrrc23	0.53010338306804
Odf2l	0.532040518648405
Adamtsl5	0.5356162705115
Irgm	0.536485471063049
Slfn9	0.538174201435255
Mx2	0.540737477181825
C1h6orf120	0.541385805480247
Hycc2	0.541428823257186
Zkscan8	0.546120067598066
Samd9	0.547060634798185
Ifitm1	0.547108014710785
Zfp77	0.548812766039329
Cobll1	0.550080213370884
Cacna1e	0.550904007871254

Matn2	0.554610602222501
Grb14	0.556579577853578
Gulp1	0.558857508919606
Kdm7a	0.559788773351398
Samd9l1	0.562860824418443
Zfp709l3	0.569159745918736
A3galt2	0.569283138974037
Abhd14a	0.571886037782545
Myo1f	0.572272208389024
Trim21	0.573420328376749
Tsx	0.585586814959888
Chic1	0.585586814959888
Zbtb40	0.587317129909373
Cybb	0.588968127735464
Abcc6	0.589553557535237
Dnah9	0.589723442997882
Ptprc	0.594002402732484
Slc25a37	0.595591499626827
Hydin	0.596515270545674
Plcg2	0.596530354394425
Nat8f5	0.597211682390627
Parp14	0.601169441623805
Tmem140	0.601806293836547
Itpr3	0.605750518153078
Slc39a12	0.607615919461711
Dcp2	0.608768055141107
Myc	0.612173480390506
Ms4a6bl1	0.617461136024158
Gbp7	0.621278572489955
Rbl1	0.623803838722272
Tnfrsf11b	0.628303098749301
Gli1	0.631395090368699
Casp1	0.631693057570704
RT1-S3	0.633167522444053
Xaf1	0.63342670439355
Slfn2	0.633882557521407
Mir100hgl	0.64082524843999
Cep295	0.642428225623659
Mzf1	0.643797867973605
Scai	0.645038961663888
Ifit1bl	0.645943822413204
Fmn1	0.646599181225977
Zfp709l1	0.647520136736067
Zfp790l3	0.647520136736067
St8sia4	0.651903863290982
Uba7	0.653179658608178
Mov10	0.662466864565191
Slc11a1	0.663143327312229
Bst2	0.665293723302391
Chrdl1	0.673702745127918
Ino80d	0.674705645125597

LOC10091120	0.67558423315163
Slc7a11	0.677794215768359
Rnf213	0.679609102537501
Cd55	0.681298628748623
Tead1	0.685686831078973
Fos	0.687893170904491
Oasl2	0.690008682072551
Rex2l3	0.697070026931251
RT1-T24-1	0.711007478908797
RT1-T24-3	0.711007478908797
Apol7a	0.719085025069167
Plekha4	0.721543080976917
Cldn19	0.722269154118315
Hic1	0.727961991052417
Nlrp1a	0.72929437036991
Agbl3	0.729742415037333
Alox5	0.731119715115504
Drp2	0.734487291120529
Fgf7	0.739020154436596
Ifi27	0.748684685529045
Znf431l-ps	0.751237574353759
RT1-T24-4	0.751581243060892
Lama3	0.754156257031034
Cdh19	0.758453733629818
2010315B03	0.767161025135402
Ifi44	0.775082956561012
Vwa3a	0.779238100528317
Hcar2	0.787635506021202
Zfp950l11	0.791914695280961
Sycp3	0.793864120892131
Zfp457	0.794376197338585
Oas1a	0.798239259060895
Ifi44l	0.800189195095293
Sbspon	0.801430809777156
Ifi47	0.807304317549611
Cxcl10	0.830373246253052
Trim34	0.833217277854316
Rsad2	0.83923681610028
Igsf7	0.843394810028308
Igsf7 l1	0.843394810028308
Tcaf2c	0.843990004331975
Knl1	0.853205428610557
Itga1	0.85546562819255
Usp18	0.862929449970007
Nlrc5	0.865651424676498
Zfp54	0.867219814367738
Zfp677	0.867219814367738
Pde4c	0.86846734997867
Nlrc4	0.872904395717787
Gas2l3	0.876314039113837
Cyp26b1	0.896990604442866

Napsa	0.898108235990368
Fxyd2	0.90350408432581
Rtp4	0.928919122280742
Lilrb3	0.940494476963168
Hemgn	0.942316113694337
Herc6	0.967260340281886
Chrm4	0.977614649074582
Myoc	0.980228389251748
Gzmm	1.01124956631487
Sostdc1	1.01756586620329
Spin2b	1.01870404022003
Rnf212	1.01980309012304
Olr59	1.03230716555789
Oas1b	1.03661254325261
Klf1	1.03906409149227
Irf7	1.04432528096776
Gimap9	1.05690731411188
Hsh2d	1.05855551031582
Gypa	1.05887783567911
Cd36	1.06199024819277
Cd36l1	1.06199024819277
Cd36-ps1	1.06199024819277
Fmo2	1.07463647296479
Efcc1	1.07613984352137
Oca2	1.08666074970831
Lrrc74b	1.09361871860029
Scn7a	1.09472777982973
Col24a1	1.10899865046329
Atp4a	1.14737110710817
Egr3	1.15279768082465
Ap1s3	1.1595753731845
Itga4	1.16779956507474
Mir770	1.17589536315571
Ppl	1.17702068375257
Siglec8	1.18417883328985
Cyp4f18	1.19306622057881
Spta1	1.20209593092143
Mx1	1.21386485981412
Sptbn5	1.22115360213491
Car1	1.22359458890517
Siglec1	1.23420514090035
B3gnt5	1.2556708787992
Msr1	1.25837449969264
Prrg4	1.25941290645663
Slfn1	1.29180007175359
Gda	1.30058022419908
Hcar1	1.31482280784267
Cd300c2	1.33184404347201
Jhy	1.33815907892182
LOC102556(	1.36292763406026
Ly6c	1.43350728353202

Jchain	1.50792764558747
Ptpn20	1.59931614477928
Oas2	1.72632333072179
Ermap	1.72765727298599
Cxcl11	1.78575268683555
Tmprss2	1.79011001104773
Pirt	1.81998130702229
Scn10a	1.91164938693614
Olr1585	1.91182773702256
Npas4	1.95157181587964
Nat8f2	1.9755846152015
Mmrn1	1.98254961149759
LOC10254790	2.20424932509865
Rhag	2.25968643702352
Ly6l	2.52674823397754
LOC12009360	2.52674823397754
Reg3b	2.64193374628439
Ccl21	2.6777787207306
LOC10255090	2.70227417149255
Cdhr3	2.71303445342136
C18h18orf6	2.89283257207843
Zfp95018	3.12709788716536
LOC690006	3.33340682256499
Htr3b	3.4681427061175
LOC362382	3.66892899513105
LOC500181	3.88923136711353
LOC690275	4.09034130701256
LOC10255280	4.09034130701256
LOC12009330	4.48630990619691
Mrgprb4	4.80833117175557

DEGs in spinal cord tissue_0.5

gene	log2FoldChange
Csn1s1	-5.288896297
Oxtr	-5.274796193
Cesl1	-4.211464995
Tspoap1	-4.096292296
Angptl4	-3.99036354
Cimap1d	-3.974223361
Klhdc7a	-3.905602764
Mup5	-3.833923052
Mup4l1	-3.833923052
Mup4l3	-3.833923052
LOC1201028	-3.833923052
Minar1	-3.717287434
Slc19a3	-3.712360408
Sucnr1	-3.625894901
Cyp2f4	-3.532676494
Ttpa	-3.515239815
Pilrb2l3	-3.394202271
Ces2b	-3.29670722
Acsbg3	-3.237135515
Mis18a	-3.055666504
Klkb1	-2.847980232
Acvr1c	-2.82332469
Chac1	-2.813209167
Irx3	-2.806855919
Pdk4	-2.792511416
Ces2g	-2.739676708
Pasd1	-2.69516897
Ankef1	-2.678978863
Grifin	-2.677117905
Mc2r	-2.67257706
Sult1e1	-2.649826317
Irx5	-2.638322511
Rhbg	-2.569460048
Ucp3	-2.563412572
Gbp6-ps2	-2.55904376
Sim1	-2.549823211
Nnat	-2.542790961
Il1r2	-2.445644117
Glt6d1	-2.444162012
Cidec	-2.429484025
Il13ra2	-2.422933332
Dlg2	-2.420674177
Gbp1	-2.354988215
Siglec10	-2.342348912
Nxph1	-2.336512688
Mal2	-2.327037725
Dnah3	-2.314693596

Spock3	-2.29825205
Rbp7	-2.259160141
Slc16a14	-2.245823788
LOC120099(	-2.183247138
LOC120101(	-2.183247138
Kcnip1	-2.142062607
Mmp3	-2.141574871
Tshr	-2.137291586
Myo7b	-2.134330171
Ptger3	-2.12508664
Rps27a-ps1	-2.110173452
C6h14orf18	-2.082929373
Mmp27	-2.062053383
Serpine1	-2.033969169
LOC120100(	-1.992089443
Kcnk3	-1.98664717
Prkar2b	-1.969820979
Ccdc13	-1.944823154
Polq	-1.934469675
Trh	-1.908300994
Aqp7	-1.901037723
Hcar1	-1.864700079
Rn5-8s	-1.861748645
Lgals12	-1.858282041
Nr5a1	-1.837551428
Slitrk4	-1.809874103
Dpep1	-1.796123862
Trarg1	-1.790115222
Erfe	-1.786593214
RGD156077	-1.783053902
Nr4a3	-1.769338415
Hhipl2	-1.764929634
Nrg4	-1.755155602
Angpt4	-1.751640435
Kcnf1	-1.733335745
Slc1a3	-1.703837552
Slc7a15	-1.654612267
Sez6l2	-1.648177084
Col26a1	-1.64137164
Mst1	-1.632517857
Arxes1	-1.626685978
Arxes2	-1.626685978
Sorcs3	-1.625710638
Kcnmb2	-1.591964794
Sele	-1.579822546
Ephx3	-1.568095529
Stc1	-1.565358382
Col1a1	-1.557873172
Grin3a	-1.550066546
Resp18	-1.549573611
Cd79a	-1.526353207

Gpr27	-1.514799518
Psat1	-1.514261695
Slc30a2	-1.513237562
Tmeff1	-1.494393322
Dpp6	-1.460305335
Pnmt	-1.453570405
Cpz	-1.428290408
Sfxn1	-1.42298159
Phgdh	-1.41349778
Il17re	-1.41163226
Neto2	-1.41084282
Fgf10	-1.404921981
Rasd1	-1.399950236
Cgref1	-1.389573729
Odaph	-1.377075388
C1qtnf6	-1.360960254
Notum	-1.354101602
Gprin3	-1.352975692
Pmfbp1	-1.350770523
Postn	-1.340064068
Adamts12	-1.334713414
Mns1	-1.332619772
Apold1	-1.325334559
Tacr1	-1.320654886
Lrrc27	-1.31780647
Sdr42e1	-1.308862469
Clcnkb	-1.305924233
Pitx2	-1.295565921
Ccdc80	-1.287410561
Prnd	-1.283852735
LOC1200939	-1.282564284
LOC1200948	-1.282564284
LOC1200954	-1.282564284
LOC1201003	-1.282564284
LOC1201012	-1.282564284
LOC1201012	-1.282564284
LOC1201032	-1.282564284
LOC1201032	-1.282564284
Col3a1	-1.275766212
Rbm3	-1.27551063
Calml3	-1.273030327
Asns	-1.255320861
Tmem132e	-1.250497546
Nr1d1	-1.245298741
Apcdd1	-1.236165489
Col4a5	-1.23451866
Fos	-1.233669067
Tdrp	-1.232309538
C19h16orf7	-1.230493041
Cacna1d	-1.230405657
Scnn1b	-1.223668401

Pi15	-1.219206587
Hoxa2	-1.219146942
Epor	-1.218047555
Dnase1l3	-1.217628059
Sema5b	-1.192865404
Lrch2	-1.186767984
Trhde	-1.184077624
Csrnp1	-1.183407047
Cep85l	-1.178401852
Rnf152	-1.174336177
Gnai1	-1.166249641
Fzd4	-1.159679065
Sparc	-1.157830135
Sbk3	-1.157103445
Gem	-1.154453479
Apln	-1.152368122
Cldn15	-1.144998547
Col4a2	-1.13826473
Emx2	-1.134816835
Col1a2	-1.127611795
Kcnk2	-1.11381736
Gpihbp1	-1.113039656
RT1-CE14	-1.111312181
Hr	-1.1108709
Ephb1	-1.110165101
Tbx1	-1.107294944
Nppc	-1.102775925
Rcan1	-1.093705801
Ccdc81	-1.092885324
Plcb1	-1.089136984
Myocd	-1.088405184
Kcnab1	-1.08177481
Qpct	-1.073258211
Zfp385a	-1.063377289
Otud1	-1.061254719
Sh3rf2	-1.04734404
Cpxm1	-1.024040255
Pparg	-1.022943116
Btla	-1.020495836
Ptprz1	-1.016595387
Col4a1	-0.987396396
Bhlhe41	-0.981408807
Inhbb	-0.98112399
Zfp622	-0.970731771
Retreg1	-0.970731771
Aldh1l2	-0.967611206
Nrip3	-0.961740448
Loxl2	-0.957769401
Cnksr2	-0.95733054
Spmip6	-0.955227138
Lox	-0.95408727

Scarb1	-0.936909944
Dusp14l1	-0.936643147
Meox1	-0.936329555
Adam12	-0.917488802
Susd4	-0.915893296
Fjx1	-0.913659432
Clec2l	-0.911775661
Fn1	-0.909045352
Cyp2j10	-0.905992653
Ankrd2	-0.905518965
Gspt2	-0.90491574
Xpnpep2	-0.903743833
Plvap	-0.902894504
Obp3	-0.897201035
Plk3	-0.896236521
Cpa1	-0.892443782
Socs3	-0.887973805
Lcn2	-0.885086015
Arap2	-0.881961512
Lhx2	-0.881846941
Fabp4	-0.881631029
Ror2	-0.878125266
Sfrp2	-0.876469274
Npr3	-0.875361017
Htr7	-0.872511117
Cdh6	-0.867967693
Megf10	-0.863321175
Cyp2j4	-0.862902074
Cyp2j3	-0.862902074
Nwd1	-0.862560081
Mmd	-0.858140593
Cldn5	-0.853293404
Dusp14	-0.84655122
Adra2a	-0.842447301
Sik2	-0.84189162
Col6a3	-0.840346583
Col4a3	-0.837872632
Clmp	-0.835311111
Adamts9	-0.831826995
Ginm1	-0.831115568
Gja1	-0.825178771
C1qtnf1	-0.825107926
Mest	-0.824859689
Sik1	-0.820146436
Spon1	-0.818675749
Mstn	-0.815270956
Tmem132a	-0.811536565
Mthfd2	-0.808522693
H3f3l1	-0.804429697
H3f3c	-0.804429697
Pnma8b	-0.802652663

Eln	-0.798317724
Nr4a1	-0.797146091
Mmp23	-0.797124614
Rn45s	-0.795885807
Acp5	-0.793554723
Igfbp3	-0.793170444
Snai3	-0.790762284
Cyb561	-0.78878223
Dpt	-0.788314882
Frem1	-0.788259953
Ntrk2	-0.788142502
Klf10	-0.783993954
C1qtnf3	-0.782519174
Cav2	-0.780780018
Klf11	-0.774571324
Gadd45b	-0.772415758
Klhl33	-0.771244694
Tmem254	-0.770876524
Synj2	-0.766704147
Dusp4	-0.762488639
Cdca7l	-0.762225573
Ptpn4	-0.76140327
Tmem178a	-0.759695692
Ampd3	-0.75907421
Tyms	-0.758175737
ATP8	-0.757568968
Eepd1	-0.755733119
Tspan12	-0.753581961
St6galnac3	-0.751014998
LOC1025545	-0.747962066
Adamts2	-0.747589456
Pcdhb9	-0.747236326
ND5	-0.745144746
Gpr88	-0.744329818
Tnfrsf11b	-0.739465057
Ppp1r3a	-0.737708205
Prr32	-0.73682568
Enc1	-0.736448263
Fbxo30	-0.735766837
Fap	-0.732299609
Car5b	-0.726851401
Plcl1	-0.725752735
F2rl1	-0.724960553
Klf9	-0.723962811
Tmem252	-0.721222509
Mfap2	-0.721205153
Ptn	-0.719857747
Pcsk5	-0.718915944
Egr1	-0.717079007
Col18a1	-0.713602534
Pot1b	-0.712949171

Kcne4	-0.712112101
Robo2	-0.706484929
Trim63	-0.706466618
Pcolce	-0.705499645
Evc2	-0.703742234
Hes1	-0.702938885
Ephx2	-0.702700553
Btnl9	-0.701577311
Col6a2	-0.698559405
Ctsk	-0.69693778
Pmaip1	-0.695777258
Hapstr1	-0.695453273
Slc8a3	-0.691566604
Tspan2	-0.691445866
ND2	-0.690708242
Fnip1	-0.68538816
Dnah11	-0.684457251
Car4	-0.683967608
S100a1	-0.683716027
Agbl1	-0.678438504
Col5a1	-0.676874946
Rapgef5	-0.675825621
Prune2	-0.675754876
Mlf1	-0.67199513
Glb1l2	-0.67172841
Ret	-0.669341364
Stk39	-0.668722966
Tbc1d15	-0.667721431
Arhgap28	-0.665453299
Arl4a	-0.665446864
Cilp	-0.662286392
Slc39a11	-0.661587753
Shox2	-0.659860644
Col5a2	-0.659837504
Olfml2b	-0.651850773
Ppic	-0.644405488
Slc20a2	-0.642182043
Slc2a13	-0.638659889
ND6	-0.637423909
Bhlhe40	-0.636995853
Ddi2	-0.636085646
Spsb4	-0.635485638
Ugp2	-0.634803692
Col6a1	-0.633015802
Strip2	-0.632486545
Endou	-0.630550965
Cdc14a	-0.630289927
C1qtnf2	-0.628557115
Rcn1	-0.6269535
Thy1	-0.62602247
Osmr	-0.62522566

Tnfaip6	-0.624006254
Fhl3	-0.622915985
Hbegf	-0.620348603
Casp12	-0.616664717
Timp3	-0.616434215
Nova1	-0.615581938
Nampt	-0.615108529
Diras3	-0.612616052
Ubtd2	-0.611536872
Papln	-0.607487082
Igf1	-0.60739808
Zfp955b	-0.605014757
Slc25a5-ps	-0.6039168
Ntng2	-0.603219844
Nrarp	-0.600579523
Agpat3	-0.600394588
Cited2	-0.600321717
COX2	-0.599905863
Fgd4	-0.598697249
Rarres2	-0.598563331
Fgl2	-0.597412024
Aebp1	-0.596958785
Adgrg2	-0.596613143
Tmem45a	-0.596397245
Slc6a9	-0.595592084
Cd248	-0.591411823
Nfatc2	-0.591378634
Ctdspl	-0.590534826
Rtn4rl1	-0.589426798
Bpnt2	-0.589355347
Alg13l1	-0.586335279
Hif1a	-0.583102509
Arc	-0.582493066
Art1	-0.581562162
Col15a1	-0.581362401
Rara	-0.578879699
Zfand5	-0.578467082
Cmpk1	-0.578005478
Atosa	-0.577924773
Acsl1	-0.577243747
Enpp2	-0.574901885
Lpin3	-0.574124265
Cadps2	-0.572548537
Sec14l5	-0.570071885
Ccnd1	-0.569965429
Ccdc6	-0.567044761
Snrk	-0.562998327
Abcb4	-0.562124566
Klhl34	-0.562099291
Dpp4	-0.561049659
Slc30a1	-0.559994796

RGD156532:	-0.555689918
Mylk4	-0.555689918
Dmxl2	-0.555199238
Mob1b	-0.554544246
Srek1ip1	-0.551676361
Tanc2	-0.549787725
Osbpl8	-0.548844727
Cmklr1	-0.545749989
C11h21orf9	-0.54466297
Prom1	-0.544452807
Fhl5	-0.543367919
Armcx4	-0.541859507
Man1a1	-0.541328989
Mnda	-0.541222778
Npnt	-0.54103738
Svep1	-0.540191284
Ankrd13c	-0.538685546
Ulk1	-0.538626136
Smc4	-0.537765819
Filip1	-0.536511294
ND4	-0.536401213
Tfrc	-0.536208175
Tmem229b	-0.535821376
Kdelr3	-0.53503868
Id4	-0.53472057
Tmem9	-0.53447838
Pdlim1	-0.533684307
LOC100911	-0.532330427
Mbtd1	-0.531856852
Arl6	-0.530855515
Plagl1	-0.53058476
ATP6	-0.530582341
Erbin	-0.528354108
Cercam	-0.527587792
Ace	-0.52662379
Golm2	-0.526413378
Pla1a	-0.525835906
Ncoa7	-0.525128738
Mtdh	-0.524194841
Mia2	-0.523827897
Yipf4	-0.523650784
Hspg2	-0.523542468
Rap1b	-0.522616007
Bcat1	-0.520724625
Itih5	-0.519885822
Twist1	-0.518765064
Tcp11l2	-0.51827266
Mgll	-0.517574582
Ccnyl1	-0.516970741
Tbcel	-0.516645799
Hey1	-0.515982788

Pdgfrl	-0.515731686
Ddx3x	-0.514722226
Npy1r	-0.514554004
Creb3l1	-0.513277048
Prkx	-0.513209822
Pm20d2	-0.512090607
Tead1	-0.511899456
Adgrg1	-0.510571446
Nat1	-0.510163515
Nat2	-0.510163515
S1pr3	-0.510010398
Lpl	-0.509160027
LOC1201024	-0.508630063
Psme2	-0.508249529
Ccnd2	-0.507980856
Pank1	-0.506997463
Frem2	-0.503838266
Cd38	-0.503729416
Serpinf1	-0.502934234
Ets2	-0.502347314
Cd24	-0.500914388
Unc93b1	0.505202271041772
Ppp1r27	0.506935245281472
C13h1orf53	0.508114816619544
Trit1	0.509870883524408
Osr1	0.509882031876259
Dnajb2	0.511869078232705
Chrna1	0.511949651128687
Ears2	0.512117308910631
Brme1	0.512246125584648
Mthfsd	0.513465288091033
Napb	0.51364517410217
LOC1009099	0.514898098507329
Ddo	0.516172941207881
Ddit3	0.516505651219113
Chchd5	0.517450411227632
Ppara	0.517645481589742
Wdr76	0.517938376883951
Stbd1	0.517991740959422
Ift27	0.518079100381654
Ptafr	0.520679781579146
Abcd1	0.523524824657211
Anks3	0.525188566966397
Med9	0.529165733888784
LOC1083483	0.53026819490398
Lyz2	0.530542919606505
Lrrc14	0.530585029151845
Zfp213	0.532212327053008
Rasgrp2	0.532598287927709
P4ha1	0.532685233888506
Hinfp	0.532840690418392

Hoxc6	0.534117105155898
Sema4a	0.534980913250155
Acox2	0.535977825306249
Tubd1	0.53653277850993
Hus1	0.537938993434777
Map3k14	0.539056386595556
Zfhx2	0.540594384412492
Kctd11	0.542094099925141
Inka2	0.542502413907544
Cry1	0.545213144542752
Wrn	0.545502331464543
Pla2g4b	0.546213847346216
Fermt3	0.546290726786407
Rhbdd2	0.547700818560006
RT1-M3-1	0.550532276963167
Lsmem2	0.551910396180694
Fzd2	0.552233976146958
Hck	0.552351275313339
Plcd4	0.55444782577924
Fcgr1a	0.556049290519445
Dync2i2	0.556309957247035
Siae	0.557226476034861
Myo1f	0.558016010310128
Nudt18	0.558434747434571
Thpo11	0.56373606995021
Rasl12	0.564933764009692
Dynll1	0.566910657152866
Mpped2	0.569572925879159
Rnpc3	0.570075226847467
Tlr2	0.572140598335894
Trpv2	0.573949292508144
Bphl	0.575252367427932
Tecpr2	0.577832639003382
Vit	0.579420883387359
Arhgap4	0.581425951280578
Igsf7	0.583387829915861
Igsf7 l1	0.583387829915861
Ovol1	0.583720338718356
Barx2	0.584193330717498
Samhd1	0.584657339468199
Ttll12	0.585397701885091
Cntn2	0.585483281826043
Serac1	0.588324048534566
Ccdc61	0.589490787379384
Ezr	0.592089073488843
Zfp112	0.59252416109891
Mid1ip1	0.593214060897233
Slc26a10	0.594873291550436
Leng8	0.595355118317941
Rtp4	0.597223372821294
Pstpip1	0.598246336330077

Zfpl	0.598393680364167
Mrm1	0.598540597659998
Slx4	0.601469123456199
Hook2	0.603197573110049
RGD1559575	0.604352540949021
Nfam1	0.604588462551541
Kctd21	0.610204945544837
Ankrd26	0.614206987158802
Hmmr	0.618144675656056
Krt10	0.618194861079218
Pak1	0.619475759341775
Dok2	0.620562768330605
Uba7	0.620568889283988
Itgb2	0.620876483123175
Tnfsf10	0.62222179998736
Slc4a4	0.626198443514012
Atg16l2	0.627756507801512
Fndc4	0.629633301210691
Xaf1	0.635192295063454
Bex1	0.638117376736426
Fgr	0.639418506452478
Ly6c	0.64471538001199
Zfyve19	0.652027134282335
Ddx60	0.653529807632345
LOC1025527	0.654779893119181
Mdc1	0.655287911606422
Myl4	0.655320147189252
Piezo2	0.656181782422509
Chrnd	0.656979720026756
Polm	0.661854852852035
Mettl7a	0.667003837131628
Poli	0.667008933486732
Traf3ip3	0.667455285458346
Nrbp2	0.668722399235922
Kcnn1	0.671892490177763
Shisa3	0.68061730176628
Kifc2	0.680662349963123
Klhl17	0.681724001784032
Exo5	0.68244628228555
Slc46a3	0.684276509029471
Zfp692	0.687008681413997
Plekhn1	0.691928389138953
Cd37	0.694368343383647
Dlg5l	0.694511840179916
Mov10	0.697064176664313
Zfp365	0.699292558836937
Prima1	0.707511189186895
Tifab	0.710363077359058
Nsun6	0.710745740699682
Rsrp1	0.712791386452258
Zfr2	0.721153562271741

Gimap7	0.738117067312659
C3ar1	0.740051532752504
Col27a1	0.742558184218963
Sh2b2	0.744040000785223
S100a9	0.745706529390756
Cd274	0.746340011336882
Rexo4	0.750295879244066
Hp	0.751639895736007
Otub2	0.753343771322972
Ifit1bl	0.755484995611689
B3gntl1	0.759724920135659
Cpxm2	0.760490040154673
Lonrf2	0.765273395369516
Msr1	0.765318795095745
Cd300c2	0.766851939610946
Gls2	0.767514347926623
Carns1	0.76840404103108
Zbtb26	0.770428189997447
Matk	0.772721570501804
Epb41l5	0.774618916503587
Shfl	0.785915952464193
Lonrf3	0.789248163606156
Morn3	0.795025354828105
Cpm	0.808003192322553
Zp2	0.809455335281912
D430041D05	0.810030468757804
Nat8b	0.811411083000516
Igdcc3	0.811483928403248
Grin3b	0.812752296777613
Itgax	0.814410139177743
Tbxas1	0.814491940881579
Hic2	0.817016512742577
Mir100hgl	0.820456923491928
Myod1	0.822917597410457
Nmrk1	0.823638782864602
Tbx19	0.836076558690166
Invs	0.838078099220461
Znf454	0.838513191086922
Znf354b	0.838513191086922
Gabrr2	0.842126561388193
Mab21l2	0.842506541157138
Dnhd1	0.842832866622311
Tspan33	0.843392946473025
LOC1025493	0.852017588738621
Ltb	0.857403447073033
Prph	0.857701404283478
Oas1i	0.861352428905805
Pilra	0.863577019556714
Ifi27l2b	0.872024898552338
Haghl	0.872136063344535
Fndc3c1	0.878915240947934

Plxna3	0.879494922798421
LOC100910[illegible]	0.883649761093642
Fmod	0.886900470237847
Fxyd7	0.888694779064419
Rnf207	0.903464995581367
Plxnc1	0.904461715121302
Igf2bp2	0.912686857166882
Kank4	0.912931347029504
Irf7	0.921936467241448
Sqor	0.926643694753643
RT1-T24-4	0.927220357954794
Fcgr2a	0.927793158878395
Itgal	0.928316597128841
Kcnip2	0.928455333354875
Cx3cr1	0.931378806864261
Cdc20	0.934609256943995
Trpc2	0.944476974126508
Lonrf1	0.945243182407886
LOC1025507[illegible]	0.945970410991649
Slc11a1	0.951952552887112
Wdr90	0.958205819146308
Sfrp5	0.962136674181924
Peli3	0.966523097774195
Hcn4	0.967902642735951
Pspn	0.968419334918595
Fcgr2al1	0.973662234316152
Fcgr2a-ps1	0.973662234316152
Oasl	0.976174978531136
Cdk5r1	0.9843975434037
Ccdc88b	0.984945360084041
Tppp2	0.990060686464932
Prss53	1.00617186858085
Cep295	1.0141948821564
Aass	1.01822603256856
Nfil3	1.02004617405324
Myog	1.02584141311887
Mir3064	1.03366594180659
Slc49a3	1.03533129081349
LOC120094[illegible]	1.03838061411364
Oas1b	1.04039325945366
Cyb5d2	1.0515624276409
Lilra5	1.05212618896046
Oasl2	1.05304446336214
Enho	1.05352937524414
Mmp11	1.06188751480308
Prf1	1.07492346046527
Siglec1	1.07946592081735
Sbspon	1.0822416170432
Rtl3	1.08350978604741
Mx2	1.08651747506534
Tmem150b	1.08999487919709

Fbln1	1.10206276101817
Gabbr2	1.10208715612923
Mybph	1.11479041697474
Atp1b4	1.11809412079537
RT1-T24-1	1.13618534571771
RT1-T24-3	1.13618534571771
Zfp286a	1.14781888742833
Ctxn3	1.14814348825229
Krt15	1.14844692169402
Krt14	1.14844692169402
Krt16	1.14844692169402
Krt19	1.14844692169402
Ka11	1.14844692169402
Mamdc4	1.14980470450286
Pilrb-ps6	1.15336979324299
Lilrb4	1.15586064962327
Aldh3a1	1.15648915371354
Siglec8	1.15859856650029
Ccdc158	1.15989589858049
Mx1	1.17096514764487
Xrcc3	1.1759379960392
Dqx1	1.19444159821361
Uchl1	1.19887903085856
Recql4	1.19931846787663
Prom2	1.20813059922708
Cited1	1.21853277977152
Septin3	1.21939243968836
Atp8b4	1.22549317779711
Scd2	1.23463089555022
Rap1gap	1.2512433949696
Cd8a	1.25566526718862
Oas1a	1.25825390487765
Zfyve28	1.25863002539666
Tlr1	1.25935263960192
Oas3	1.26391227648605
Cyp2s1	1.27335967019953
Abcg4	1.27482008787682
Dusp15	1.30899581571738
Csf3r	1.32845030344232
Tmprss5	1.32968573579372
Ankrd1	1.33577177527049
LOC102551[illegible]	1.35695548968246
Cntfr	1.35726684698425
Gzmm	1.36951219701215
Actc1	1.37530196018984
Dmpk	1.37730365440425
Wnt6	1.38434024746635
Ccdc78	1.4021897267177
Tnnt2	1.41538119041772
Fcnb	1.43237112266843
Efna2	1.44842007766571

Prss35	1.45179603227976
Adgrg5	1.46770160826262
Grhl2	1.47021577171682
Cd5	1.49398628725283
Ppfia3	1.49420515055858
Gzmc	1.49750958598987
Gzmb	1.49750958598987
LOC1003612	1.49750958598987
LOC1025538	1.49750958598987
Mag	1.49826167088299
Slc51a	1.51158333576426
Slc28a1	1.52885076196817
Fam83g	1.53069509279694
Il2rb	1.53764025444282
Ggt6	1.54943938702407
Adgre4	1.61075476532557
Cyp26b1	1.63773869473853
Edaradd	1.65443166372586
Rhox5	1.65620847191125
Spem1	1.69106716925044
Arhgef19	1.6919852941526
Comp	1.74527282763029
Wdr97	1.76297463401903
Mir27b	1.82581210608934
H2bc18	1.85812898842721
Paqr5	1.87108869963276
Lilrb3	1.88137073328814
Nmrk2	1.88989363086576
Oas2	1.90510825500375
Sln	1.91107329376893
C2h1orf162	1.95802573450514
Slitrk3	2.03921147602614
G0s2	2.04188213075335
Dnah9	2.08017799322531
Syngr3	2.13934990040233
Far2	2.14474108959965
Gpr21	2.21687698658541
Znf750	2.27747387868654
Zfp683	2.27762556599251
Gpr35	2.28368215761955
Zic1	2.28898205638903
Gtf2a1l	2.48207849512749
Eps8l1	2.54789760822603
Clec4e	2.61117195055183
Hpd	2.68832549721335
Retnlg	2.70416546164901
Slc25a43	2.71483536502551
Cd8b	2.78110774863375
Coch	2.78698375173054
Cbln2	3.38626874787374
LOC690499	3.40826779168243

LOC690509	3.40826779168243
Il21	3.50233685168418
Ccl1	3.61352249815408
Mir1b	3.70952485992079
C19h4orf51	3.80055733684081
Olr1877	4.0875458326849
Foxd1	4.12807064880583
Arhgef39	4.46107870626012
LOC1036909	4.46845444400022
Tnn	4.51988757210682

Co-DEGs

gene	
Rbp7	down
Pilrb2l3	down
Mis18a	down
Fabp4	down
Postn	down
Apold1	down
Polq	down
Eln	down
Cyp2j10	down
Sema5b	down
Ephx2	down
Ginm1	down
Nat8b	up
Arhgap4	up
Invs	up
Rnpc3	up
Leng8	up
Mx2	up
Myo1f	up
Dnah9	up
Xaf1	up
Mir100hg	up
Cep295	up
Ifit1bl	up
Uba7	up
Mov10	up
Slc11a1	up
Oasl2	up
RT1-T24-	up
RT1-T24-	up
RT1-T24-	up
Oas1a	up
Sbspon	up
Igsf7	up
Igsf7 l1	up
Cyp26b1	up
Rtp4	up
Lilrb3	up
Gzmm	up
Oas1b	up
Irf7	up
Siglec8	up
Mx1	up
Siglec1	up
Msr1	up
Cd300c2	up
Ly6c	up
Oas2	up
